# Supplementary material for: Effect of metformin on insulin resistance in adults with type 1 diabetes: a 26-week randomized double-blind clinical trial
Source: Nat Commun. 2025 Nov 24;16:9884. doi: 10.1038/s41467-025-65951-1 (PMC12644478; doi:10.1038/s41467-025-65951-1)
Supplement: Supplementary file 1 — Supplementary Information [file 41467_2025_65951_MOESM1_ESM.pdf]

SUPPLEMENTARY INFORMATION

Effect of Metformin on Insulin Resistance in Adults with Type 1 Diabetes: a 26-week Randomized Double-blind Clinical Trial (the INTIMET Study)

Table S1. The contributions of steady-state glucose and insulin to hyperinsulinemic-euglycemic clamp variables per low and high-dose insulin phases……………………………………………………………………………2

Table S2. Relationships between insulin resistance and cardiometabolic measures …….3

Table S3. The impact of acute or chronic glycemia on glucose infusion rate (muscle insulin sensitivity). ……...…4

Table S4. The effect of metformin and placebo on primary and secondary metabolic endpoints, from model-predicted treatment estimates. …………………………………………………………………………………….5

Table S5. Effect of metformin on insulin resistance measures and total daily insulin dose after adjustment for factors. ………8

Table S6. The effect of metformin and placebo on continuous glucose monitoring metrics..……………………….…9

Table S7. The effect of metformin on exploratory endpoints; glucagon and dietary intake………………………10

Table S8. Safety outcomes by treatment group…………………………………………………………………12

Trial Protocol…………………………………………………………………………………………...13

Statistical Analysis Plan………………………………………………………………………………...49

Table S1. The contributions of steady-state glucose and insulin to hyperinsulinemic-euglycemic clamp variables per low and high-dose insulin phases.

Low-dose insulin phase insulin and glucose levels did not significantly impact EGP and NEFA measurements, yet high-dose insulin phase insulin concentration contributed to GIR prediction, supporting the need for adjustment of this covariate (Table A). Participants with type 1 diabetes remained insulin resistant at liver, adipose and muscle even after adjusting for glucose and insulin (Table B).

A.

	Insulin concentration during clamp			Glucose concentration during clamp		
Dependent variable	β coefficient	Std error	p-value	β coefficient	Std error	p-value
LD-EGP (µmol/kgFFM/min)	-0.009	0.0058	0.10	0.209	0.182	0.25
LD-NEFA (mmol/L)	0.00004	0.0008	0.96	0.004	0.025	0.87
HD-GIR (µmol/kgFFM/min)	-0.188	0.09	0.03	4.8	8.1	0.56

B.

	Unadjusted model		Model adjusted for insulin and glucose		
Dependent variable	Type 1 diabetes	Control	Type 1 diabetes	Control	Adjusted model p-value
LD-EGP (µmol/kgFFM/min)	5.9 ± 2.2	3.6 ± 1.7	5.9 ± 0.3	3.6 ± 0.5	0.0003
LD-NEFA (mmol/L)	0.08 ± 0.01	0.02 ± 0.02	0.08 ± 0.01	0.01 ± 0.02	0.005
HD-GIR (µmol/kgFFM/min)	61.9 ± 20.1	87.7 ± 18.4	61.5 ± 2.8	89.4 ± 4.0	0.00000002

LD-EGP low-dose insulin phase endogenous glucose production; LD-NEFA low-dose insulin phase non-esterified fatty acids; HD-GIR high-dose insulin phase glucose infusion rate. Unadjusted data presented as mean ± SD and mean ± SEM for models adjusted for insulin and glucose concentration during clamp using generalized linear modelling. Analyses are two-sided, without adjustment for multiple comparisons.

Table S2. Relationships between insulin resistance and cardiometabolic measures

The inverse of the glucose infusion rate (GIR) from the high-dose clamp stage represents muscle insulin resistance, and endogenous glucose production (EGP) during the low-dose clamp stage represents liver insulin resistance.

	Correlation to muscle insulin resistance (1/GIR)		Correlation to liver insulin resistance (low-dose EGP)	
Metabolic factor	r	p-value	r	p-value
Whole cohort (with and without type 1 diabetes)				
sICAM-1 (ng/mL)	0.402	0.002	0.302	0.02
SE-selectin (ng/mL)	0.349	0.007	0.313	0.02
Log-AIx (%)	0.258	0.048	0.156	0.22
BMI (kg/m^2)	0.304	0.02	0.172	0.19
VAT (g)	0.269	0.04	0.06	0.65
ALT (IU/L)	0.289	0.03	0.110	0.41
GGT (IU/L)	0.408	0.01	0.215	0.10
ALP (IU/L)	0.288	0.03	0.389	0.002
LDL (mmol/L)[#]	0.322	0.024	-0.076	0.60
Type 1 diabetes only				
HbA1c (%)	0.329	0.04	0.307	0.06
TDI (units/kg/day)	0.524	0.0009	0.239	0.15
Daytime glucose (mmol/L)	-0.209	0.22	0.162	0.35
Overnight glucose (mmol/L)	0.330	0.05	0.188	0.27
CGM CV (%)	-0.489	0.002	0.201	0.24
Fasting glucose (mmol/L)	0.095	0.56	0.057	0.73

AIx augmentation index, BMI body mass index, VAT visceral adipose tissue, sICAM-1 soluble intercellular adhesion molecule 1, sE-selectin soluble endothelial selectin, ALT alanine transaminase, GGT gamma-glutamyltransferase, ALP alkaline phosphatase, low-density lipoprotein, HbA1c Hemoglobin A1c, TDI total daily insulin dose, CGM CV coefficient of variation. [#]Statins were excluded for LDL analysis only. Pearson and Spearman's correlation were used according to normality of the data. These analyses are two-sided, and data are presented without correction for multiple testing, as these analyses are exploratory.

Table S3. The impact of acute or chronic glycemia on glucose infusion rate (muscle insulin sensitivity).

Fasting glucose level at the time of commencement of the hyperinsulinemic-euglycemic clamp did not significantly impact GIR measurements, yet adjustment for HbA1c attenuated the between group difference between participants with and without type 1 diabetes.

	Dependent variable: GIR (µmol/kgFFM/min)						
	Fasting glucose on day of clamp				HbA1c		
Parameter	β coefficient	Std error	p-value	Parameter	β coefficient	Std error	p-value
Intercept	69.4	11.2	0.0000000006	Intercept	110.9	24.4	0.000005
Type 1 diabetes*	22.2	7.4	0.03	Type 1 diabetes*	10.4	9.2	0.26
Fasting glucose	-0.7	1.1	0.48	HbA1c	-6.6	3.3	0.04

*type 1 diabetes = 1, control without diabetes =0

HbA1c; glycated hemoglobin, HD-GIR high-dose insulin phase glucose infusion rate. Analyses are two-sided using generalized linear modelling without adjustment for multiple testing.

Table S4. The effect of metformin and placebo on primary and secondary metabolic endpoints, from model-predicted treatment estimates.

	Metformin				Placebo				Estimated treatment difference		
Primary endpoint	Baseline	26 weeks	Modelled difference (95% CI)	p-value	Baseline	26 weeks	Modelled difference (95% CI)	p-value	Model factors	Beta coefficient (95% CI)	p-value
EGP (µmol/kgFFM/min)	6.0 ± 2.4	5.7 ± 1.8	-0.3 (-1.5 to 0.9)	0.66	5.8 ± 2.0	5.4 ± 1.7	-0.4 (-1.6 to 0.9)	0.54	Group*time<br>Time	0.2 (-0.4 to 0.8)<br>-0.4 (-1.3 to 0.5)	0.53<br>0.36
Secondary endpoints											
GIR (µmol/kgFFM/min)	62.4 ± 20.9	71.9 ± 18.9	8.3 (0.8 to 15.9)	0.03	61.3 ± 19.7	66.8 ± 20.5	6.6 (-1.2 to 14.6)	0.10	Group*time<br>Time	1.6 (-4.8 to 7.9)<br>6.7 (0.3 to 13.0)	0.62<br>0.04
NEFA (mmol/L) ^	0.04 (0.06)	0.02 (0.05)	-0.02 (-0.07 to 0.03)	0.38	0.06 (0.12)	0.04 (0.10)	-0.02 (-0.06 to 0.02)	0.41	Group*time<br>Time	-0.12 (-0.5 to 0.3)<br>-0.4 (-0.8 to -0.05)	0.54<br>0.03
BMI (kg/m^2)	27.0 ± 3.7	26.5 ± 3.3	-0.1 (-0.5 to 0.3)	0.59	25.8 ± 3.8	25.9 ± 4.3	0.3 (-0.1 to 0.7)	0.13	Group*time<br>Time	-0.3 (-0.8 to 0.2)<br>0.2 (-0.1 to 0.6)	0.27<br>0.21
Weight (kg)	81.4 ± 12.5	81.2 ± 13.2	-0.3 (-1.5 to 1.0)	0.67	77.5 ± 12.6	78.6 ± 14.1	1.0 (-0.3 to 2.2)	0.12	Group*time<br>Time	-0.9 (-2.6 to 0.7)<br>0.8 (-0.4 to 2.0)	0.27<br>0.18
Waist (cm)	89.5 ± 8.2	89.4 ± 7.0	1.0 (-0.6 to 2.6)	0.23	87.8 ± 12.0	90.8 ± 13.9	3.0 (1.3 to 4.7)	0.001	Group*time<br>Time	-1.4 (-3.6 to 0.6)<br>2.7 (1.1 to 4.3)	0.16<br>0.001
WHR	0.88 ± 0.08	0.89 ± 0.08	0.01 (-0.02 to 0.03)	0.57	0.90 ± 0.08	0.93 ± 0.09	0.04 (0.01 to 0.07)	0.007	Group*time<br>Time	-0.02 (-0.05 to 0.01)<br>0.04 (0.01 to 0.06)	0.06<br>0.004
Blood pressure<br>Systolic (mmHg)	117 ± 13	115 ± 7	-1.9 (-6.3 to 2.6)	0.41	121 ± 12	117 ± 8	-4.3 (-8.9 to 0.2)	0.06	Group*time<br>Time	-0.9 (-4.3 to 2.4)<br>-2.6 (-6.2 to 1.0)	0.58<br>0.16
Diastolic (mmHg)	73 ± 7	71 ± 7	-2.3 (-5.2 to 0.6)	0.12	73 ± 8	72 ± 7	-0.7 (-3.7 to 2.2)	0.62	Group*time<br>Time	-0.6 (-2.9 to 1.6)<br>-1.2 (-3.6 to 1.2)	0.58<br>0.32
Glycemia and insulin<br>HbA1c (%)	7.4 ± 0.6	7.2 ± 0.7	-0.2 (-0.4 to 0.1)	0.15	7.6 ± 1.1	7.4 ± 0.9	-0.2 (-0.4 to 0.1)	0.12	Group*time<br>Time	-0.04 (-0.3 to 0.2)<br>-0.2 (-0.4 to 0.1)	0.77<br>0.14
Insulin dose (units/kg/day)	0.56 ± 0.13	0.52 ± 0.14	-0.03 (-0.082 to 0.025)	0.29	0.71 ± 0.23	0.72 ± 0.23	0.04 (-0.01 to 0.10)	0.14	Group*time<br>Time	-0.10 (-0.15 to -0.04)<br>0.1 (0.01 to 0.1)	0.0008<br>0.024
Insulin dose (units/day)	45.3 ± 12.3	42.6 ± 13.0	-2.2 (-6.4 to 2.0)	0.30	55.6 ± 23.1	55.4 ± 23.4	3.0 (-1.3 to 7.4)	0.17	Group*time<br>Time	-6.5 (-11.4 to -1.7)<br>3.7 (-0.3 to 7.6)	0.009<br>0.07

Mean glucose (mmol/L)	9.3 ± 1.6	9.3 ± 1.4	-0.01 (-0.5 to 0.0.5)	0.96	9.6 ± 2.0	9.1 ± 1.6	-0.4 (-1.0 to 0.2)	0.19	Group*time<br>Time	0.1 (-0.4 to 0.7)<br>-0.3 (-0.8 to 0.2)	0.57<br>0.30
Mean overnight glucose (2400–0600) (mmol/L)	8.9 ± 2.1	9.2 ± 1.7	0.3 (-0.4 to 1.0)	0.39	9.5 ± 2.3	8.8 ± 1.7	-0.6 (-1.4 to 0.3)	0.18	Group*time<br>Time	0.2 (-0.4 to 0.9)<br>-0.2 (-0.9 to 0.5)	0.47<br>0.55
Mean daytime glucose (0600-2400) (mmol/L)	9.5 ± 1.6	9.4 ± 1.6	-0.1 (-0.7 to 0.4)	0.64	9.7 ± 1.9	9.3 ±1.8	-0.3 (-1.0 to 0.4)	0.38	Group*time<br>Time	0.1 (-0.5 to 0.6)<br>-0.2 (-0.8 to 0.3)	0.84<br>0.39
CV (%)	36.6 ± 7.4	36.9 ± 6.2	0.1 (-2.8 to 3.0)	0.95	35.4 ± 5.8	34.8 ± 5.5	-0.6 (-2.9 to 4.0)	0.74	Group*time<br>Time	0.9 (-1.2 to 3.1)<br>-0.7 (-3.3 to 1.8)	0.39<br>0.58
Body composition total fat mass (g) ^	25534 ± 10741	24517 ± 10732	-224 (-1144 to 697)	0.63	22627 ± 7315	23550 ± 7848	421 (-494 to 1337)	0.36	Group*time<br>Time	-0.02 (-0.07 to 0.03)<br>0.01 (-0.03 to 0.05)	0.49<br>0.48
total fat free mass (g)	55806 ± 9413	55074 ± 9955	-78 (-809 to 652)	0.83	55075 ± 10093	55244 ± 10833	169 (-603 to 940)	0.66	Group*time<br>Time	-207 (-1237 to 822)<br>148 (-611 to 908)	0.69<br>0.70
visceral adipose tissue (g) ^	421 (542)	352 (375)	-15.2 (-88.6 to 58.2)	0.68	529 (971)	719 (1038)	94.2 (-208 to 397)	0.54	Group*time<br>Time	-0.3 (-0.7 to -0.02)<br>0.3 (0.04 to 0.5)	0.04<br>0.02
CAP score (dB/m) ^	222 ± 32	228 ± 42	5.5 (-14.7 to 25.6)	0.59	238 ± 41	229 ± 62	-11.4 (-33.1 to 10.4)	0.30	Group*time<br>Time	0.0003 (-0.06 to 0.06)<br>-0.01 (-0.1 to 0.06)	0.99<br>0.75
Mean anterior thigh muscle fat infiltration (%)^	4.0 ± 1.3	4.2 ± 1.4	0.2 (0.04 to 0.4)	0.02	4.0 ± 0.8	4.2 ± 0.9	0.1 (-0.3 to 0.1)	0.25	Group*time<br>Time	0.03 (-0.03 to 0.10)<br>0.03 (-0.01 to 0.07)	0.31<br>0.17
Abdominal subcutaneous adipose tissue volume (L) ^	6.3 (6.3)	5.3 (3.3)	-0.21 (-0.67 to 0.26)	0.38	4.4 (3.3)	5.0 (3.8)	-0.051 (-0.375 to 0.272)	0.75	Group*time<br>Time	-0.007 (-0.1 to 0.1)<br>-0.02 (-0.1 to 0.1)	0.89<br>0.57
Liver fat (%)^	1.5 (0.6)	1.1 (0.9)	-0.3 (-0.8 to 0.3)	0.35	1.9 (1.9)	2.1 (1.7)	-0.1 (-0.6 to 0.8)	0.82	Group*time<br>Time	-0.3 (-0.6 to 0.1)<br>-0.001 (-0.3 to 0.3)	0.11<br>0.99
Blood metabolites<br>Adiponectin (µg/mL) #	1.8 ± 0.7	1.7 ± 0.7	-0.1 (-0.3 to 0.1)	0.51	1.6 ± 0.7	1.7 ± 0.7	0.1 (-0.1 to 0.3)	0.29	Group*time<br>Time	-0.1 (-0.1 to 0.1)<br>-0.02 (-0.1 to 0.1)	0.89<br>0.57
Total cholesterol (mmol/L) ^	4.4 ± 0.8	4.1 ± 0.9	-0.2 (-0.5 to 0.03)	0.08	4.2 ± 0.8	4.2 ± 0.9	-0.004 (-0.3 to 0.3)	0.97	Group*time<br>Time	-0.03 (-0.1 to 0.03)<br>-0.02 (-0.1 to 0.04)	0.41<br>0.54
LDL (mmol/L) ^	2.7 ± 0.7	2.4 ± 0.8	-0.2 (-0.4 to -0.01)	0.04	2.4 ± 0.6	2.4 ± 0.6	0.01 (-0.2 to 0.2)	0.92	Group*time<br>Time	-0.04 (-0.1 to 0.04)<br>0.02 (-0.1 to 0.04)	0.32<br>0.48
HDL (mmol/L) ^	1.3 ± 0.3	1.3 ± 0.3	-0.1 (-0.2 to 0.04)	0.21	1.4 ± 0.5	1.4 ± 0.4	0.04 (-0.1 to 0.1)	0.51	Group*time<br>Time	-0.05 (-0.1 to 0.03)<br>0.01 (-0.1 to 0.1)	0.18<br>0.7
Cholesterol/HDL ratio^	3.4 ± 0.6	3.4 ± 0.9	-0.03 (-0.4 to 0.3)	0.88	3.3 ± 0.8	3.0 ± 0.9	-0.3 (-0.6 to 0.03)	0.08	Group*time<br>Time	0.1 (-0.02 to 0.2)<br>-0.1 (-0.2 to -0.003)	0.13<br>0.04
Triglycerides (mmol/L) ^	0.84 ± 0.41	0.96 ± 0.57	0.1 (-0.01 to 0.3)	0.07	0.81 ± 0.35	0.79 ± 0.28	-0.03 (-0.2 to 0.1)	0.69	Group*time<br>Time	0.1 (-0.05 to 0.3)<br>-0.03 (-0.1 to 0.2)	0.28<br>0.68

GDF15 (pg/mL)^	553 (204)	858 (662)	399 (93 to 704)	0.01	641 (214)	549 (351)	17 (-102 to 136)	0.78	Group*time<br>Time	0.3 (0.1 to 0.4)<br>0.1 (-0.02 to 0.3)	0.001<br>0.09
Inflammatory and vascular<br><br>Augmentation index (AIx)	10.8 ± 11.1	9.3 ± 9.0	-1.6 (-5.1 to 1.8)	0.35	11.6 ± 11.9	10.9 ± 10.7	-1.3 (-4.9 to 2.3)	0.48	Group*time<br>Time	-0.5 (-3.8 to 2.8)<br>-1.2 (-4.3 to 1.8)	0.76<br>0.43
Exploratory											
sICAM1 (ng/mL)	214.4 ± 47.5	203.0 ± 52.6	-10.4 (-20.7 to -0.3)	0.04	213.4 ± 49.1	211.5 ± 58.7	-5.1 (-15.5 to 5.4)	0.34	Group*time<br>Time	-4.0 (-16.1 to 8.0)<br>-5.8 (-15.3 to 3.7)	0.51<br>0.23
SE-selectin (ng/mL)	34.6 ± 14.4	34.5 ± 14.9	1.0 (-0.8 to 2.9)	0.27	35.4 ± 14.5	34.8 ± 14.7	-0.8 (-2.7 to 1.2)	0.44	Group*time<br>Time	1.5 (-1.0 to 4.0)<br>-0.6 (-2.5 to 1.2)	0.22<br>0.51
IL-6 (pg/mL)^	1.2 ± 0.6	1.4 ± 0.8	0.1 (-0.1 to 0.4)	0.30	1.2 ± 0.7	1.3 ± 0.9	0.1 (-0.1 to 0.3)	0.46	Group*time<br>Time	0.1 (-0.1 to 0.2)<br>0.1 (-0.1 to 0.2)	0.44<br>0.47
Uric acid (mmol/L)	0.2 ± 0.1	0.3 ± 0.1	0.02 (0.001 to 0.034)	0.04	0.3 ± 0.1	0.3 ± 0.1	0.004 (-0.014 to 0.022)	0.65	Group*time<br>Time	0.003 (-0.02 to 0.02)<br>0.009 (-0.01 to 0.03)	0.73<br>0.24
IGF-1 (nmol/L)	18.7 ± 5.1	17.8 ± 3.4	-0.2 (-1.6 to 1.2)	0.80	19.2 ± 4.4	19.6 ± 4.8	0.8 (-0.6 to 2.2)	0.28	Group*time<br>Time	-0.8 (-2.1 to 0.6)<br>0.7 (-0.5 to 1.9)	0.27<br>0.26

Baseline and 26-week data are descriptive summaries of the raw data, expressed as mean ± SD or median (interquartile range). Treatment difference values are modelled estimated means and 95% CI from the intention-to-treat population. Estimated treatment differences are baseline-adjusted, using generalized linear mixed modelling with the interaction of treatment group and time as the fixed effect (group*time) and the individual participant as the random effect. Analyses are two-sided and without adjustment for multiple testing. ^indicates that the model required a log-link function, and the estimated treatment difference is presented on a log-scale. #Adiponectin was input as log-adiponectin as the response variable. A small constant (half of the smallest non-zero value) was added for models with a log-link function and datapoints that included values with zeros.[13] To convert the values for HbA1c to mmol/mol, multiple the value by 10.93 then subtract 23.5.

BMI body mass index, CAP controlled attenuation parameter, CV coefficient of variation, EGP endogenous glucose production, HbA1c hemoglobin A1c, GDF15 growth differentiation factor 15, GIR glucose infusion rate, HDL high density lipoprotein, IGF-1 insulin-like growth factor-1, IL-6 interleukin-6, LDL low density lipoprotein, NEFA non-esterified fatty acids, sICAM1 soluble intercellular adhesion molecule 1, sE-selectin soluble endothelial selectin, WHR waist-hip-ratio

Table S5. Effect of metformin on insulin resistance measures and total daily insulin dose after adjustment for factors.

Adjust for age, sex, baseline BMI, baseline HbA1c and insulin delivery did not alter the effect of metformin on insulin resistance measures, compared to unadjusted models reported in Table S4. There was a significant effect of metformin on insulin dose compared to placebo after adjustment for these factors.

	Unadjusted model			Adjusted model		
Dependent variable	Model factors	Beta coefficient (95% CI)	p-value	Model factors	Beta coefficient (95% CI)	Adjusted model p-value
EGP (µmol/kgFFM/min)	Group*time	0.2 (-0.4 to 0.8)	0.53	Group*time	0.3 (-0.3 to 0.9)	0.39
	Time	-0.4 (-1.3 to 0.5)	0.36	Time	-0.5 (-1.4 to 0.4)	0.27
				Age	0.03 (-0.03 to 0.09)	0.39
				Sex	-0.03 (-1.1 to 1.1)	0.96
				Baseline BMI	-0.01 (-0.2 to 0.1)	0.88
				Baseline HbA1c	0.7 (0.1 to 1.3)	0.02
				Insulin delivery method	-0.2 (-1.2 to 0.7)	0.63
GIR (µmol/kgFFM/min)	Group*time	1.6 (-4.8 to 7.9)	0.62	Group*time	2.6 (-3.2 to 8.5)	0.38
	Time	6.7 (0.3 to 13.0)	0.04	Time	6.2 (0.002 to 12.4)	0.05
				Age	-0.3 (-0.9 to 0.4)	0.42
				Sex	0.05 (-12.0 to 12.1)	0.99
				Baseline BMI	-2.1 (-3.7 to 0.6)	0.008
				Baseline HbA1c	-3.7 (-10.2 to 2.8)	0.26
				Insulin delivery method	-7.6 (-18.2 to 3.1)	0.16
NEFA (mmol/L)^	Group*time	-0.12 (-0.5 to 0.3)	0.54	Group*time	-0.2 (-0.6 to 0.2)	0.35
	Time	-0.4 (-0.8 to -0.05)	0.03	Time	-0.4 (-0.8 to 0.01)	0.04
				Age	0.01 (-0.04 to 0.05)	0.77
				Sex	0.32 (-0.4 to 1.1)	0.42
				Baseline BMI	0.15 (0.04 to 0.2)	0.01
				Baseline HbA1c	0.1 (-0.3 to 0.6)	0.54
				Insulin delivery method	0.2 (-0.5 to 0.9)	0.59
Total daily insulin dose (units/kg/day)	Group*time	-0.10 (-0.15 to -0.04)	0.0008	Group*time	-0.1 (-0.2 to -0.05)	0.0005
	Time	0.1 (0.01 to 0.1)	0.024	Time	0.06 (0.01 to 0.1)	0.02
				Age	-0.001 (-0.01 to 0.01)	0.78
				Sex	0.07 (-0.1 to 0.2)	0.32
				Baseline BMI	0.01 (-0.004 to 0.03)	0.14
				Baseline HbA1c	0.05 (-0.02 to 0.1)	0.19
				Insulin delivery method	-0.004 (-0.1 to 0.1)	0.94

BMI body mass index; HbA1c; glycated hemoglobin, EGP low-dose insulin phase endogenous glucose production; NEFA low-dose insulin phase non-esterified fatty acids; GIR high-dose insulin phase glucose infusion rate. Data presented as baseline-adjusted modelled estimated treatment difference and 95% CI from the intention-to-treat population with the interaction of treatment group and time (group*time), and modelled factors as fixed effects and the individual participant as the random effect. ^indicates that the model required a log-link function, and the estimated treatment difference is presented on a log-scale. A small constant (half of the smallest non-zero value) was added for models with a log-link function and datapoints that included values with zeros.[13] Sex (male = 1, female = 0), insulin delivery method (pump = 1, multiple daily injections = 0), Analyses are two-sided and without adjustment for multiple testing.

Table S6. The effect of metformin and placebo on continuous glucose monitoring metrics.

Baseline and post-treatment values are modelled estimated means (95% CI) from the intention-to-treat population. The treatment effect was estimated using generalized linear mixed modelling (beta binomial models) and is expressed as odds ratio (OR) of time spent in glucose range category. Analyses are two-sided and without adjustment for multiple testing.

	Baseline		26 weeks		Treatment effect	
	Metformin	Placebo	Metformin	Placebo	Odds ratio (95% CI) (reference is placebo*time)	p-value
% time 3.9-10mmol/L (70-180mg/dL)	59.6 (50.8 to 67.8)	60.9 (52.0 to 69.2)	58.4 (49.5 to 66.8)	61.9 (52.4 to 70.5)	0.92 (0.3 to 1.3)	0.66
% time < 3 mmol/L (55 mg/dL)	0.768 (0.397 to 1.48)	0.504 (0.233 to 1.09)	0.549 (0.261 to 1.15)	0.480 (0.205 to 1.12)	0.75 (0.8 to 2.2)	0.59
% time <3.9 mmol/L (70 mg/dL)	2.65 (1.64 to 4.26)	1.94 (1.15 to 3.24)	2.49 (1.49 to 4.13)	2.66 (1.56 to 4.5)	0.68 (0.2 to 1.2)	0.16
% time >10 mmol/L (180mg/dL)	35.4 (26.6 to 45.4)	34.9 (25.9 to 45.0)	37.3 (28.1 to 47.5)	33.3 (24.1 to 43.9)	1.2 (0.6 to 1.8)	0.50
% time >13.9 (>250 mg/dL)	10.4 (6.0 to 17.3)	10.1 (5.7 to 17.2)	8.8 (4.9 to 15.3)	7.0 (3.7 to 12.8)	1.3 (0.9 to 2.5)	0.52

Table S7. The effect of metformin on exploratory endpoints; glucagon and dietary intake

	Metformin				Placebo				Estimated treatment difference		
Endpoint	Baseline	26 weeks	Difference (95% CI)	p-value	Baseline	26 weeks	Difference (95% CI)	p-value	Model factors	Beta coefficient (95% CI)	p-value
Glucagon measures											
Fasting glucagon (ng/mL)^	16.2 (7.1 to 36.8)	15.7 (6.9 to 35.7)	-0.5 (-4.1 to 3.1)	0.78	13.6 (6.0 to 31.0)	14.1 (6.2 to 32.2)	0.5 (-2.7 to 3.7)	0.75	Group*time<br>Time	-0.05 (-0.1 to 0.2)<br>0.02 (-0.1 to 0.2)	0.57<br>0.80
Glucagon during low-dose clamp phase (ng/mL)^	6.9 (2.8 to 17.0)	5.5 (2.2 to 13.6)	-1.4 (-3.1 to 0.3)	0.11	6.3 (2.5 to 15.7)	5.3 (2.1 to 13.1)	-1.0 (-2.5 to 0.5)	0.18	Group*time<br>Time	-0.04 (-0.2 to 0.2)<br>-0.2 (-0.4 to 0.04)	0.97<br>0.02
Diet measures											
Energy intake (calories/day)	1837 (1090 to 2584)	1773 (1025 to 2520)	-64 (-222 to 93)	0.42	1789 (1042 to 2536)	1750 (1002 to 2498)	-38 (-196 to 118)	0.62	Group*time<br>Time	-2 (-146 to 150)<br>-52 (-186 to 81)	0.98<br>0.44
Carbohydrate intake (g/day)	176 (88 to 263)	174 (86 to 261)	-2 (-22 to 17)	0.82	164 (76 to 252)	166 (78 to 253)	2 (-18 to 21)	0.86	Group*time<br>Time	-2 (15 to 20)<br>-1 (-18 to 15)	0.79<br>0.87
Protein intake (g/day)	88 (44 to 132)	86 (42 to 130)	-2 (-12 to 9)	0.72	91 (47 to 135)	84 (40 to 128)	-7 (-17 to 4)	0.20	Group*time<br>Time	2 (-7 to 11)<br>-5 (-14 to 4)	0.72<br>0.24

Fat intake (g/day)	78 (37 to 119)	74 (33 to 115)	-3 (-13 to 6)	0.47	75 (34 to 116)	74 (33 to 115)	-1 (-11 to 8)	0.79	Group*time<br>Time	-0.3 (-9 to 8)<br>-2 (-10 to 6)	0.95<br>0.58
Sugar intake (g/day)	66 (20 to 111)	64 (18 to 109)	-2 (-13 to 9)	0.71	62 (16 to 108)	62 (16 to 108)	0.3 (-10 to 11)	0.95	Group*time<br>Time	0.1 (-9 to 9)<br>-1 (-10 to 8)	0.98<br>0.85

Data are mean (95% CI). Baseline and post-treatment values are modelled estimated means from the intention-to-treat population. Estimated treatment differences are baseline-adjusted, using generalized linear mixed modelling with the interaction of treatment group and time as the fixed effect (Group*time) and the individual participant as the random effect. Analyses are two-sided and without adjustment for multiple testing. ^indicates that the model required a log-link function, and the estimated treatment difference is presented on a log-scale.

Table S8. Safety outcomes by treatment group

Safety or adherence outcome	Metformin N=20	Placebo N=20
Adverse events (n)	3	2
Participants (n,%)	2 (10)	2 (10)
Serious adverse events (n)	2	0
Participants (n,%)	1 (5)	0
Non serious adverse events (n)	1	2
Participants (n,%)	1 (5)	2 (10)
Related events		
Gastrointestinal event	0 (0)	2 (10)
DKA event	0 (0)	0 (0)
Severe hypo event	0 (0)	0 (0)
Unrelated events		
Hospitalisation for presyncope	1 (5)	0
Hospitalisation for appendicitis	1 (5)	0
Iron deficiency	1 (5)	0
Medication adherence (% target)	89	88

Serious adverse event was defined as death, is life-threatening, necessitate hospitalisation or results in permanent disability. Related and unrelated adverse events were defined as according to whether they were deemed by the study investigator to have reasonable possibility of an association with the study drug.

Medication adherence was assessed by tablet return (total tablets divided by treatment days, divided by target dose, times 100). Tablet count data was available for n=15 placebo, n=17 metformin participants who attended their final visit. DKA; diabetic ketoacidosis.

INTIMET Study Protocol

Insulin Resistance in Type 1 Diabetes Managed with Metformin

A Detailed Study of Liver and Muscle Insulin Resistance: Hyperinsulinaemic-Euglycaemic Clamps to Assess the Efficacy of Adjunctive Metformin in Adults with Type 1 Diabetes

Sponsor: Garvan Institute of Medical Research

Project Team Roles and Responsibilities

Study Coordinator: Dr Jennifer Snaith

[redacted]

Responsibilities: Coordination of study, conduct experiments and collate, analyse and publish data.

Principal Investigator: Professor Jerry Greenfield

[redacted]

Responsibilities: PhD supervisor to Jennifer Snaith (primary supervisor), clinical trial design

Principal Investigator (SVH Site): Associate Professor Mark Danta

[redacted]

Responsibilities: advice regarding protocol design and result interpretation (specifically fibroscans), supervision of study activities at SVH

Principal investigator (Westmead Site): A/Prof Jane Holmes-Walker

[redacted]

Responsibilities: PhD supervisor (secondary supervisor), clinical trial design

Co Investigator: Dr Christian Girgis

[redacted]

Responsibilities: PhD supervisor (secondary supervisor), clinical trial design

[redacted]

Co Investigator: Dr Dorit-Samocha Bonet

[redacted]

Responsibilities: advisory to clinical trial design and protocol

Associate Investigator: Professor Paul Mitchell

[redacted]

Responsibilities: advice regarding protocol design, conducting ophthalmologic investigations

Associate Investigator: Associate Professor Andrew White

[redacted]

Responsibilities: advice regarding protocol design and interpretation

Associate Investigator: Associate Professor Clinton Bruce

[redacted]

Responsibilities: advice regarding insulin clamp design, sample analysis and interpretation

Associate Investigator: Dr Greg Kowalski

[redacted]

Responsibilities: advice regarding insulin clamp design, sample analysis and interpretation

Associate Investigator: Dr Hamish Dunn

[redacted]

Responsibilities: advice regarding protocol design, interpretation of ophthalmologic investigations

Summary

**Study Title**	<u>Short name</u>:<br>INTIMET – Insulin Resistance in Type 1 Diabetes Managed with Metformin<br><u>Detailed name</u>:<br>Hyperinsulinaemic-Euglycaemic Clamps to Assess the Efficacy of Adjunctive Metformin in Adults with Type 1 Diabetes: a randomised placebo-controlled trial and detailed study of liver and muscle resistance
**Objectives**	<u>Primary</u><br>Main endpoint is improvement in hepatic insulin resistance.<br><u>Secondary</u><br>In adults with T1D, to assess the effect of metformin on:<br>• Peripheral insulin resistance<br>• diabetes measures (HbA1c, glycaemic variability, time in range, time in hypoglycaemia, total daily insulin dose, total daily bolus dose, total daily basal dose)<br>• diabetes complications (albuminuria, retinopathy)<br>• anthropometrics (BMI, weight, waist circumference, waist:hip, blood pressure)<br>• hormones and metabolites (lipid profile, adiponectin, GDF15, SHBG etc)<br>• gastrointestinal microbiota<br>• body composition (liver fat, total fat mass, total free-fat, visceral fat, bone density)<br>• liver stiffness<br>• indices of oxidative and inflammatory stress (c-reactive protein, E-selectin ICAM-1, IL-6, arterial stiffness radial artery applanation)<br>• bone health (turnover, density)<br>• mitochondrial action<br>We will also assess if the site of insulin resistance (liver vs muscle vs both) has an adverse effect on the above parameters.
**Study design**	2 part multi-centre randomised placebo-controlled study<br><u>Part 1</u><br>Cross-sectional study to quantify, phenotype and determine the pattern of insulin resistance in adults with T1D and adults without diabetes as a comparator group (2:1 ratio).<br><u>Part 2 (main study)</u><br>Double blinded randomised placebo-controlled trial of 6 months of metformin XR therapy (1.5g daily) in adults with T1D (1:1 ratio)
**Planned sample size**	60 participants (40 T1D, 20 without diabetes)
**Selection criteria**	<u>Inclusion Criteria</u>

	• Age range: 20-55 years (and pre-menopausal if female)<br>• Type 1 diabetes:<br>o Disease duration > 10 years (ie. chronic exogenous insulin exposure)<br>o Insulin deficiency (fasting c-peptide < 0.3nmol/L)<br>• Willingness to consent, participate and comply with the study<br><u>Exclusion Criteria</u><br>• HbA1c > 9.5% (to avoid confounding by chronic glucotoxicity affecting insulin resistance)<br>• Current smoking<br>• Medications that affect glucose metabolism (glucocorticoids, antipsychotics, immunosuppressants). These also cannot be given during the clinical study.<br>• Exposure to metformin within the last 30 days<br>• Alcohol intake > 20g/day in women or > 40g/day in men (to exclude ethanol effect on liver)<br>• Weight change > 5% in last 3 months or history of bariatric surgery<br>• Pregnancy, breastfeeding, or childbearing potential not willing to avoid pregnancy during the study<br>• Known major organ dysfunction (eGFR < 60, liver disease transaminases x3 upper limit of normal, cardiac event within the last 6 months, current cancer or uncontrolled thyroid dysfunction). These conditions either interfere with the excretion or metabolism of the test medication or would interfere with measurement of the study outcome.<br>• Recent episode of diabetes ketoacidosis or severe hypoglycaemia (hypoglycaemia requiring assistance) in the last 6 months. Exclusion is in view of known counter-regulatory hormone effects on insulin resistance.<br>• A history of a psychological illness or condition such as to interfere with the patient's ability to understand the requirements of the study.
**Study procedures**	<u>Part 1: Characterisation Phase (T1D and non-diabetes)</u><br>Visit 1: DXA scan, fibroscan, accelerometer, abdominal MRI. Diet history, DXA, (and insulin dose diary and CGM for T1D)<br>Visit 2A: insulin clamp study, urine sample, blood samples, stool sample, oral swab, tonometry, calorimetry<br>Visit 2B: retinal assessment (optional)<br><br><u>Part 2: Intervention Phase (T1D only – following 6 months metformin 1.5g daily)</u><br>3-month review: phone call, blood sample, stool and oral sample<br>Visit 3: DXA scan, fibroscan, accelerometer, abdominal MRI. Diet history, DXA, insulin dose diary and CGM<br>Visit 4A: insulin clamp study, urine sample, blood samples, stool sample, oral swab, tonometry, calorimetry

	Visit 4B: retinal assessment (optional)<br>3-months after study drug last dosed: stool and oral sample<br>Visit 5: pQCT (optional)
**Statistical considerations**	Analysis plan: Mann-whitney and t-tests, linear regression analysis, generalised linear mixed modelling
**Study duration**	3 years

TABLE OF CONTENTS

1. BACKGROUND 10
1.1. DISEASE BACKGROUND 10
1.2. RATIONALE FOR PERFORMING THE STUDY 10
2. STUDY OBJECTIVES 11
2.1. PRIMARY OBJECTIVE 11
2.2. SECONDARY OBJECTIVES 12
3. STUDY DESIGN 12
3.1. DESIGN 12
3.2. STUDY GROUPS 12
3.3. NUMBER OF PARTICIPANTS 13
3.4. NUMBER OF SITES 13
3.5. DURATION 13
4. PARTICIPANT SECTION 13
4.1. INCLUSION CRITERIA 13
4.2. EXCLUSION CRITERIA 13
5. STUDY OUTLINE* 14
5.1. STUDY FLOW CHART 14
5.2. INVESTIGATION PLAN 15
5.3. STUDY PROCEDURE RISKS 23
5.4. RECRUITMENT AND SCREENING 25
5.5. INFORMED CONSENT PROCESS 26
5.6. ENROLMENT PROCEDURE 26
5.7. RANDOMISATION PROCEDURE 27
6. TISSUE COLLECTION/BIOBANKING 27
7. SAFETY 28
7.1. ADVERSE EVENT REPORTING 29
7.2. SERIOUS ADVERSE EVENT REPORTING 29
7.3. DATA SAFETY AND MONITORING BOARD 29
7.4. EARLY TERMINATION 29
8. BLINDING AND UNBLINDING 29
9. OUTCOMES AND FUTURE PLANS 30
10. STATISTICAL CONSIDERATIONS 31
11. CONFIDENTIALITY AND STORAGE AND ARCHIVING OF STUDY DOCUMENTS 31
12. OTHER STUDY DOCUMENTS 33
13. RESOURCES 33
14. REFERENCES 35

1. BACKGROUND

1.1. Disease Background

Introduction:

Type 1 diabetes (T1D) affects 120 000 individuals in Australia and the incidence is rising (1). It is a condition typified by autoimmune destruction of pancreatic beta cells leading to insulin deficiency. Insulin deficiency gives rise to hyperglycaemia, which is damaging to large and small blood vessels leading to complications. Persons with T1D have an increased cardiovascular (CV) risk and excess mortality relative to the normal population. However, this increased risk is not explained by hyperglycaemia alone.

It is now understood that insulin deficiency is not the only metabolic disturbance in T1D. Insulin resistance is an under recognised but key component to the metabolic milieu in T1D and is relevant to altered pathways in fat, liver, muscle and other tissues. The Garvan laboratory has previously established that T1D individuals are more insulin resistant than their non-diabetic peers. This was determined using insulin clamp studies, the gold standard measure of insulin resistance (2). However, there remain key unanswered questions with regards to insulin resistance in T1D, including its cause, its consequences and if there is phenotypic heterogeneity within T1D populations.

Insulin clamp studies involve delivery an infusion of insulin to a participant. The sensitivity of the participant to insulin is determined by the amount of glucose required to maintain euglycaemia. It is not known if insulin resistance in T1D typically arises from liver, muscle or both and if there are inter-individual variations in organ patterns of insulin resistance. Heterogeneity in the degree and organ source of insulin resistance may explain diversity in metabolic phenotypes in T1D, where some individuals are predisposed to metabolic syndrome with weight gain, increased insulin requirements, adverse lipid and blood pressure profiles and increased cardiovascular risk. Insulin resistance may be the explanation for increased cardiovascular risk in T1D in general, and more so in some individuals. This is also strongly suggested by the finding that T1D patients with greater clamp-derived insulin resistance associate with greater degrees of coronary artery calcification (3). Heterogeneity in insulin resistance may also explain findings of variable responses to metformin in T1D.

Metformin is an insulin sensitising agent that improves glycaemia in type 2 diabetes (T2D) by lowering hepatic glucose output, and increasing peripheral glucose uptake in muscle (4). It is thought to be beneficial to CV outcomes in T2D (5). Treatments traditionally reserved for the treatment of type 2 diabetes (T2D) may serve a purpose in T1D. Disappointingly, metformin studies in T1D have not demonstrated consistent results, and at most have shown only minor improvements in weight, cholesterol, glycaemia and no clear reduction in CV risk. Insulin replacement has long been the mainstay of its treatment, but there has been little consideration regarding use of adjunctive agents to reduce insulin resistance. If it is established that metformin is beneficial to lowering insulin resistance in T1D, then treatment paradigms will change.

1.2. Rationale for Performing the Study.

Need for a trial:

There are still vast knowledge gaps in the understanding of the role and pathogenesis if insulin resistance in T1D. It has been established that T1D individuals are globally more insulin resistant than normal individuals, but the organ source of insulin resistance (muscle vs liver vs both) has not been established. There are knowledge gaps with regards to inter-individual variation in patterns of insulin resistance and if this has consequences for lipid and blood pressure profiles, weight, or cardiovascular risk.

Our trial is the first study to examine organ specific patterns of insulin resistance in T1D and is the first to study heterogeneity in insulin resistance and response to metformin.

Therefore in context of the lack of current evidence, this trial is valuable and essential. It has the potential to change treatment paradigms with an increased focus on treatment of insulin resistance in T1D. Through a better understanding of T1D pathophysiology, it will also assist in the development of future new therapeutic agents.

2. STUDY OBJECTIVES

<u>The overall aim of this project is to:</u>

1. Characterise patterns of insulin resistance in muscle and liver in T1D and determine the impact on metabolic phenotype.
2. Determine predictors of response to metformin in T1D.

<u>Specific aims include:</u>

1. To determine whether muscle and/or liver insulin resistance is present universally or selectively in people with T1D.
2. To determine whether organ specific patterns of insulin resistance in T1D cluster with adverse metabolic and cardiovascular risk factors, and bone parameters (density and turnover)
3. To identify non-invasive clinically available markers that identifies individuals with T1D with increased insulin resistance.
4. To determine the relationship between organ specific insulin resistance and response to metformin in T1D.
5. To create predictive algorithms using non-invasive measures that identify individuals with T1D with increased insulin resistance, and individuals likely to respond to metformin

<u>Hypotheses</u>:

Main hypothesis: Treatment with metformin will improve insulin sensitivity in T1D in participants displaying hepatic insulin resistance.

We also predict that:

1) In T1D, there are heterogeneous patterns of insulin resistance in muscle and liver.
2) More insulin resistant individuals will display worse metabolic and cardiovascular profiles (higher blood pressure, adverse lipid profile, visceral and hepatic steatosis).

<u>What this study will achieve:</u>

1. This study will repurpose metformin for use in T1D, with personalised prescription to those expecting greatest benefit. Through responder stratification, precision medicine can be achieved.
2. Provide immediate clinical impact – as metformin is an affordable and readily available drug.
3. Change treatment paradigms - with an increased focus on treatment of insulin resistance in T1D
4. Assist in development of new therapeutic agents through better understanding of T1D pathophysiology.

2.1. PRIMARY OBJECTIVE

The primary endpoint is change in hepatic insulin resistance.

2.2. SECONDARY OBJECTIVES

In adults with T1D, to assess the effect of metformin on:

- Peripheral insulin resistance
- diabetes measures (HbA1c, glycaemic variability, time in range, time in hypoglycaemia, total daily insulin dose, total daily bolus dose, total daily basal dose)
- diabetes complications (albuminuria, retinopathy)
- anthropometrics (BMI, weight, waist circumference, waist:hip, blood pressure)
- hormones and metabolites (lipid profile, adiponectin, GDF15, SHBG etc)
- gastrointestinal microbiota
- body composition (liver fat, total fat mass, total free-fat, visceral fat, bone density)
- liver stiffness
- indices of oxidative and inflammatory stress (c-reactive protein, VCAM-1, ICAM-1, arterial stiffness radial artery applanation)
- bone health (turnover, density)
- mitochondrial action

We will also assess if the site of insulin resistance (liver vs muscle vs both) has an adverse effect on the above parameters

2.3. STUDY DESIGN

This multicentre clinical trial has a 2-part design:

Part 1:

Observational cross-sectional study of the prevalence and pattern of insulin resistance in adults with T1D. This will be compared with a non-diabetes comparator group (2:1 ratio).

Part 2 (main study):

Double blinded randomised placebo-controlled trial of 6 months of metformin therapy (1.5g daily) in adults with T1D (1:1 ratio). The primary endpoint is change in clamp-derived insulin resistance.

The study design has been developed in accordance with:

- SPIRIT statement for interventional trials: http://www.spirit-statement.org/
- CONSORT guidelines for randomised trials: http://www.consort-statement.org/
- STROBE criteria for observational studies - https://www.strobe-statement.org

2.4. STUDY GROUPS

There will be 2 study groups.

1. Adults with type 1 diabetes –40 participants will be studied
    a. 20 will be randomised to metformin
    b. 20 will be randomised to placebo
2. Adults without diabetes – 20 participants will be studied

Recruitment criteria are further detailed in section 4.

2.5. NUMBER OF PARTICIPANTS

Sixty participants in total are required (40 with T1D and 20 without diabetes). Refer to section 10 for power calculation that provided the rationale for this sample size.

2.6. NUMBER OF SITES

Multiple centres.

Participants will be recruited from Westmead Hospital, St Vincent's Hospital and the private consulting rooms of Endocrinologists. It is expected that 30% of participants will come from each site. The main study visits (60 participants in total) will be conducted at the Garvan Institute of Medical Research and St Vincent's Hospital. Retinal assessment (optional) will be conducted at the St Vincent's Hospital Diabetes Centre. The final optional visit is at Royal North Shore Hospital. Consent will be performed at the Garvan Institute, or over the telephone, and documented electronically through RED-cap software.

2.7. DURATION

Expected study duration: 3-4 years

Start date: August 2019

End date: Feb 2022

Recruitment: start November 2019, anticipate 12-18 months of recruitment

3. PARTICIPANT SECTION

3.1. INCLUSION CRITERIA

- Sex: men and women
- Age range: 20-55 years (and pre-menopausal if female)
- Type 1 diabetes:
  - Disease duration > 10 years (ie. chronic exogenous insulin exposure)
  - Insulin deficiency (fasting c-peptide < 0.3nmol/L)
- Willingness to consent, participate and comply with the study

3.2. EXCLUSION CRITERIA

- HbA1c > 9.5% (to avoid confounding by chronic glucotoxicity affecting insulin resistance). A test within the last 3 months is adequate for entry.
- Current smoking
- Current or plan for medications that affect glucose metabolism (glucocorticoids, antipsychotics, immunosuppressants). These also cannot be administered during the clinical study.
- Exposure to metformin within the last 30 days
- Alcohol intake > 20g/day in women or > 40g/day in men (to exclude ethanol effect on hepatic steatosis)
- Weight change > 5% in last 3 months or history of bariatric surgery
- Pregnancy, breastfeeding, or childbearing potential not willing to avoid pregnancy during the study
- Known major organ dysfunction (eGFR < 60, liver disease transaminases > 3 times the upper limit of normal, cardiac event within the last 6 months, current cancer or uncontrolled thyroid dysfunction). These conditions either interfere with the excretion or

metabolism of the test medication, or would interfere with measurement of the study outcome.

- Diabetic ketoacidosis or severe hypoglycaemia (hypoglycaemia requiring assistance) in the last 6 months. This exclusion is in view of known counter-regulatory hormone effects on insulin resistance.
- Patients with a history of a psychological illness or condition such as to interfere with the patient's ability to understand the requirements of the study.

4. STUDY OUTLINE

4.1. STUDY FLOW CHART

Telephone Screening
- Pass screening: proceed to eligibility evaluation
- Not eligible: FINISH

PICF for pre-enrolment blood tests
- PICF sent. Study explained over phone
- Willing subjects: sign screening PICF and send to Garvan

Pre-enrolment blood tests
- Eligible: continue. Invited to Visit 1.
- Ineligible: FINISH

Enrolment, Baseline Visit 1 (+ Randomisation of T1D)
- Physical consent
- Visit 1 – accelerometer, DXA, Fibroscan, MRI, CGM (T1D)

**Visit 2A**<br>• Main study visit – clamp, blood, stool, tonometry, calorimetry<br>• No diabetes – FINISH<br>• T1D – start intervention (placebo vs metformin)	**Visit 2B (option)**<br>• Retina review

Monitoring 3 month blood test

Visit 3
- Accelerometer, DXA, Fibroscan, CGM

**Visit 4**<br>• Main study visit – clamp, blood, stool, tonometry, calorimetry	**Visit 4B (option)**<br>• Retina review
**Data Analysis**<br>Construction of algorithm predicting response to metformin	**Visit 5 (option)**<br>• pQCT

4.2. INVESTIGATION PLAN

Study activity	All participants			T1D participants							All
Visit	Screening	Visit 1	Visit 2	Start study drug	Phone contact	3-month contact	Visit 3	Visit 4	Phone contact	3-month post treatment contact	Visit 5 (optional)
Week		-2	-1	0	1	13	25	26	27	39	N/A
Eligibility assessment	x										
Informed consent		x									
Accelerometer		x					x				
CGM[†]		x					x				
DXA scan		x					x				
Fibroscan		x					x				
Abdominal MRI		x					x				
Retinal examination (optional)		x					x				
Exam and history			x					x			
+/- Urine ACR[†]			x								
Blood samples			x			x		x			
Stool sample			x			x		x		x	
Oral swab			x			x		x		x	
Tonometry			x					x			
Calorimetry			x					x			
Insulin clamp study			x					x			
Provide study drug[†]			x								
Adverse event assessment[†]					x	x		x	x		
pQCT											x

[†]T1D only.

Abbreviations: CGM, continuous glucose monitoring; ACR, albumin-creatinine-ratio, DXA: dual energy x-ray absorptiometry, MRI: magnetic resonance imaging, pQCT: peripheral quantitative computed tomography

Methodology:

- Physical examination:
    - Blood pressure: will be measured in a supine position after a 5 minute rest using a mercury sphygmomanometer
    - Weight: measured to the nearest 0.1kg in a hospital gown
    - Height: measured to nearest 0.01m by stadiometer with the participant barefoot
    - Waist circumference: measured as the narrowest circumference between the lowest aspects of the ribs and anterior superior iliac crests
- Detailed history:
    - Record family history

    - Record dietary composition using the Easy Diet Diary app for 7 days (then analysed using software (Xyris).
    - Physical activity questionnaire – Stanford Physical Activity Questionnaire
- Insulin clamps: 2 step hyperinsulinaemic-euglycaemic clamp with deuterated glucose tracers
    - Hyperinsulinaemic euglycaemic clamps are the gold standard for assessment of the pharmacodynamics properties of insulin, and are the best method for answering our study question.
    - It will be performed at the Garvan Institute using facilities that have performed many clamp studies over several decades. The facility is well equipped with experienced staff, protocols and necessary equipment.
    - Participants are advised 48 hours prior to the study to avoid strenuous exercise, caffeine and alcohol (to avoid consequent changes to insulin resistance). They will attend at 0800AM after a 10-hour fast. Participants with T1D will be asked to omit their morning insulin dose. They will be provided advise regarding safe management of their insulin overnight and on the morning of the study, and have the option of phone contact with study investigator and Endocrinologist (Dr Jennifer Snaith) the night before the study for assistance with this.
    - There are 2 different insulin infusion protocols that study different insulin resistance parameters. These include:
        - Low dose insulin infusion
        - High dose insulin infusion
    - Using sterile technique, 2 cannula will be inserted into each antecubital vein. One will be used to infuse insulin and dextrose. The other will be used for blood sampling. The insulin infusion uses neutral insulin (Actrapid) and the dextrose infusion uses deuterated glucose tracers [6,6-$2H_2$], 25% dextrose enriched to 2.5% with deuterated glucose).
        - The clamp starts with a 2-hour primed (5 mg/kg), continuous (3 mg/kg.h) infusion of [6,6-$2H_2$] glucose, followed by a 2-hour infusion of low-dose insulin (20 mU/m^2.min) and a 2-hour infusion of high-dose insulin (60mU/m2.min) as previously described (6).
        - The deuterated glucose infusion rate is halved (1.5 mg/kg.h) during, and ceased at the end of, the low-dose insulin infusion.
        - Glucose is infused to maintain whole-blood concentration of 5.5mmol/L with variable rate infusion of dextrose.
        - The low and high glucose infusion rates (GIR[HI]) are calculated at 90–120 minutes of each clamp stage and normalized for fat-free mass (FFM). Whole-body energy expenditure and respiratory quotient (RQ) are measured at baseline and during the last 30 minutes of each stage of the clamp (Parvo Medics True One).
        - GIR(HI): glucose infusion rate (during high-dose insulin)
            - This reflects peripheral (muscle) insulin sensitivity because endogenous glucose production (from liver) is suppressed during high dose insulin infusion Target measure: steady state glucose infusion rate (GIR). It is calculated as the mean of the last 4 recorded rates of glucose infusion during the last 30 minutes of the study.
        - Endogenous glucose production (EGP): is calculated by measuring glucose appearance and disappearance using modeling calculations.
            - estimated using Steele's one-compartment, fixed-volume model (assuming volume of distribution of 20% of body weight and pool fraction of 0.65 [9]), as modified by Finegood et al (7).

Systemic glucose appearance and disappearance are estimated using non-steady-state calculations (7).

  - At the completion of the study, the cannulas are removed, and the participant is able to eat.
- Calorimetry
  - Continuous indirect calorimetry will measure energy expenditure (EE) and a respiratory quotient (RQ), which is the ratio of carbon dioxide production to oxygen consumption. Metabolic rate is proportional to gas exchange.
  - Participants will wear a hood over their head that samples expired air. This is done twice over two 25 minute periods. The first period is before the start of the insulin infusion whilst the participant is lying flat and has rested for 20 minutes. The second period is in the last 25 minutes of the clamp study. The mask is not uncomfortable.
- DXA scan:
  - A DXA scan (dual-energy x-ray absorptiometry) will measure body composition, including total body fat (TBF), visceral adipose tissue (VAT), lean body mass, free fat mass (FFM) and bone density.
  - The radiation dose used by DXA is small (0.001mSV) which is less than a standard chest x-ray. The participant is required to lie flat on the scanner bed. The scan takes 10- 15 minutes to perform.
- Fibroscan
  - A fibroscan is a non-invasive device that assesses liver stiffness by transient elastography (by measuring the velocity of a vibration wave generated on the skin). It is also used to produce a Controlled Attenuation Parameter (CAP) score that is used to measure hepatic steatosis.
  - It is performed with the patient supine. An ultrasound-like probe is placed on the skin over the liver. The participant will not feel discomfort, but will feel a gentle vibratory sensation. It takes 10 minutes to perform.
- Abdominal MRI
  - Abdominal MRI scans provide detailed assessment of abdominal fat, liver fat and thigh fat. This will supplement the liver fat assessment provided by the fibroscan. There is no radiation exposure, and the scan takes approximately 10 minutes to perform.
  - It is performed with the patient supine on the scanner bed and does not cause discomfort.
- CGM:
  - Participants with diabetes will be fitted with a continuous glucose monitor (Medtronic iPro, or Dexcom G6), the results of which are blinded to the patient. It is a small device that is inserted into the abdominal skin, which measures interstitial glucose continuously. This will provide information about blood glucose variability, and will detect any episodes of hypoglycaemia. The device is fixed to the skin with a dressing. In the cohort provided the Medtronic CGM system, the CGM will be removed after 1 week at a subsequent study visit. A second sensor device will then be placed for a second week of monitoring. In the cohort provided the Dexcom CGM system, the CGM will be placed during the first visit, and removed at the second visit. There is minimal discomfort to the participant, asides from the time of insertion. The discomfort of this is similar to the sensation of an insulin injection. Participants are able to engage in usual activities whilst wearing the device. Up to two weeks of wear is required to capture minimum useful data.
- Accelerometer:
  - An accelerometer is a waist worn device (Actigraph GT1X) that can quantify a participant's activity levels (sedentary, light intensity, moderate intensity,

vigorous) and count steps. It is worn during waking times only for 3 to 7 days. It is comfortable to wear and will not impair the participant's daily activities. This information will be used to take into account activity levels and any correlations with insulin sensitivity.

- Tonometry:
  - Applanation tonometry is a non-invasive measure of arterial stiffness which is an independent predictor of cardiovascular mortality (8). This technique can be used to easily measure radial artery arterial stiffness and has been used at the Garvan in previous studies (9). A transducer is placed over the radial artery, compressing but not occluding the artery against the underlying radius bone. The transducer provides a waveform as well as readings of systolic and diastolic blood pressure. The technique does not cause any discomfort to the participant, asides from a feeling of gentle pressure. The technique takes 1-2 minutes.
- Blood sample:
  - There are various hormones, metabolites and markers that are of interest in our study. These markers provide information regarding diabetes control, metabolic status, and endothelial health. They will form the basis of our analysis regarding metabolic and vascular health. These markers are easily measured through a blood test and include:
  - Hormones and metabolites: HbA1c, fasting glucose, insulin, c-peptide, lipid profile (LDL, HDL, triglycerides), NEFA (non esterified fatty acids), growth hormone, IGF1, uric acid, sex hormone binding globulin, GDF15
  - Fat derived hormones: adiponectin
  - Vascular markers:
    - markers of endothelial function: plasma von willebrand factor, VCAM-1, e-selectin, t-PA, PAI-1
    - markers of inflammatory activity: CRP (+/- hsCRP), ICAM-1, IL-6
  - Bone turnover markers: CTx (crosslinked c-telopeptide), P1NP (procollagen type 1 N propeptide), OC (osteocalcin)
  - Metformin level: will be taken in T1D participants at visit 4 following 6 months of metformin treatment. The result will be used to determine if response variability has any individual pharmacokinetic basis.
  - mitochondrial action
- Urine sample:
  - A urine sample (<50mL) will be used to analyse the urine-albumin-creatinine ratio, which is a measure of proteinuria and marker of diabetic nephropathy, a complication of diabetes.
  - Urine beta-hcg testing will also be performed to rule out pregnancy at various time points including before each clamp study, and each scan involving ionising radiation.
- Stool sample:
  - A stool sample will be collected by participants at home the day before the nominated study visit. They will be provided a specialised kit. The sample will be stored for possible future analysis, such as analysis of microbiome signature and metabolome. There is current research interest regarding this topic at the Garvan Institute. Specifically for this project, gut microbiome composition analysis may provide key information regarding individual susceptibility to disease, complications and identify individuals likely to respond to metformin.
- Oral swab:
  - An oral swab will be collected at home (after provision of a collection kit), or at the Garvan institute, depending on the stage of the clinical trial. The swab will be brushed over buccal mucosa, then stored for future analysis, including

profiling of oral microbiome species. This will be correlated with the stool microbiome species, providing further information regarding upper GI tract microbiome species. This will complete the picture regarding the GI tract microbiome.

- pQCT assessment
  - peripheral quantitative CT assessment (pQCT) is an imaging technique that can make quantitative measurements of bone mineral density at a peripheral site (wrist or ankle) and is a useful measure of bone strength. The pQCT machine at Royal North Shore Hospital has been used for various research studies with radiation safety precautions that have satisfied the RNSH ethics committee. It is performed with the patient lying flat for up to 1 hour. It does not cause any discomfort.
- Retinal assessment
  - Various assessments will occur during the participant's retinal assessment visit.
  - Pupillometry (RETeval) is measured using a simple handheld device that provides quantifiable pupil reactivity data as well as flicker ERG (electroretinography) information. It does not require pupil dilation and is not invasive. Pupillometry and ERG is a sensitive measure of autonomic dysfunction, and has been used as a technique in diabetic neuropathy assessments. A small sensor strip is placed on the skin over the zygomatic bone. The device is then held over the eye and creates flashes of light. The sensor strip will detect electrical responses produced by the retina.
  - Retinal photographs will be taken with a non-mydriatic retinal camera (not requiring eye drops)
  - Images will be captured and saved via PDF, then analysed by investigator Dr Hamish Dunn.
  - Any newly detected vision threatening retinopathy will be referred to Sydney Eye Hospital for review.

<u>Visits in Detail: (see study outline 5.1)</u>

1. Advertising
   - Volunteers will be recruited from diabetes clinics and from the general public. Advertisements will be placed on notice boards in the St Vincent's Hospital precinct, and within diabetes centre notice boards and physician clinics around Sydney.
   - The study will be advertised on social media forums (on pages frequented by individuals with type 1 diabetes)
2. Pre-enrolment screening and consent
   - Location: phone call or email
   - Duration: 10 minutes
   - Detail:
     - Participants that respond to advertisements will be screened over the phone using a prepared telephone script and questionnaire (see appendix document Phone Script_Screening Questions). Participants that appear eligible will be invited to review the PICF and perform screening blood tests. They will be sent a PICF by email or post. The research coordinator will call, email or SMS participants a week later to discuss the study by phone and answer any questions arising. The method of contact will be clarified with participants at the time of first contact during telephone screening. If willing to undergo the blood tests, the participants will be asked to sign the consent and email/post back to the Garvan Institute, or will deliver the signed consent form at the time of first study visit, or will consent electronically via RED-cap software.
     - If eligible by blood tests, participants will be invited to the Garvan Institute for an Enrolment and Randomisation visit.

- A letter to GP and the participant's regular endocrinologist will be provided explain the study, and also to caution regarding avoiding contraindicated medications (if possible). See appendix Letter Notification to Physicians
- see point 5.6 for further detail regarding the recruitment and enrolment process

3. Enrolment visit and randomisation (Visit 1)
   - Location: Garvan Institute, St Vincent's Hospital, St Vincent's Clinic
   - Duration: 1-2 hour (DXA scan 10 minutes, CGM placement 20 minutes, accelerometer provision 10 minutes, fibroscan 20 minutes, MRI 30 minutes)
   - Detail:
     - Participants will be greeted 30 minutes prior to their scheduled DXA scan appointment to sign the study consent form, address any questions and perform urine bHCG testing if female. The DXA scan is at St Vincent's Clinic.
     - They will then have fibroscan testing and MRI scanning also at St Vincent's hospital. They will need to be fasting for this scan.
     - After the scans, participants will return to the Garvan Institute for placement of the CGM device (T1D only) and will be provided an accelerometer. They will wear both for 1 week.
     - Finally, instructions regarding avoidance of alcohol and strenuous exercise prior to visit 2 will be provided, as well a stool collection kit. They will record their dietary intake using an app for the week leading up to their next study visit, and in the 48 hours prior to visit 2 will be instructed to ensure at least 50% of their dietary intake is carbohydrate based.
     - If necessary, instructions regarding insulin dosing, clamp preparation, use of diet diary and accelerometer may be provided over the phone, or via tele-conferencing
     - Participants will be randomised to placebo or metformin using minimisation (Minitable program) modified from Saghaei et al (10). Factors were weighted for importance and included BMI (40% weighting), HbA1c (20% weighting), gender (20% weighting), age (20% weighting).
4. Visit 2A
   - Location: Garvan Institute
   - Duration: full day visit
   - Detail:
     - 48 hours before (at home): participants will avoid alcohol consumption and strenuous exercise. In this period, their diet will consist of at least 50% carbohydrates. 24 hours prior to visit, stool will be collected using the provided kit.
     - Participants will arrive at 0800AM after a 10 hour fast and will be asked to dress in a hospital gown. Expectations and risks of the procedure will be discussed (though participants will have had previous explanation and written material previously made available)
     - Stool sample will be stored, oral swab collected and CGM device removed (in T1D)
       A spot urine sample will be taken, and females will have a urine beta-hcg test to exclude pregnancy.
     - A detailed personal and family history, and complete diet and physical activity questionnaires (20 minutes). Physical examination will be performed including measurement of blood pressure, height, weight and waist circumference. (15 minutes)
     - Radial artery tonometry (10 minutes)
     - Insulin clamp study (up to 6 hours)
     - Indirect calorimetry (25-30 minutes on 3 occasions during the clamp study)
     - At the conclusion of the clamp, patients will be offered food.

        - They will be fitted with their second CGM device that they will wear for 1 further week (if Medtronic CGM). Participants with T1D will be provided with supply of the study intervention drug (metformin vs placebo), and an information sheet. The drug will commence after the second week of CGM has completed.

Participants without diabetes have no further visits. Subsequent visits are T1D only.

5. Visit 2B (optional)
    - Location: St Vincent's Hospital Diabetes Centre
    - Duration: 0.5 hour
    - Detail:
        - Participants will first have visual acuity assessment using a standardised Snellen chart.
        - ERG and pupillometry are performed with the pupil undilated.
        - Retinal photographs will be taken using a non-mydriatic retinal camera.
        - Standard mydriatic eye drops may be instilled into both eyes if pupil constriction limits adequate assessment of the retina. The effect is transient and usually lasts up to 4 hours. Eye drops allow for more detailed assessment of the retina without obstruction from a constricted pupil. They may cause mild stinging lasting a few seconds.
        - Reports will be uploaded as a PDF document to redcap and reviewed by study investigator Dr Hamish Dunn.
        - Vision threatening retinopathy will be referred to Sydney Eye Hospital.
6. Phone call follow up
    - Location: phone call
    - Duration: 5 minutes
    - Detail: A phone call will be made 2 weeks after participants begin study treatment to assess tolerance, adverse effects, and allow for dose adjustment.
7. 3 month contact
    - Location: phone call, blood test (external), stool and oral swab sample (home collection)
    - Duration: 5 minutes
    - Detail: 3 months after starting treatment, participants will be contacted by phone call to assess for adverse effects. They will be posted a blood test form to repeat an HbA1c, and collection kits for stool and oral swabs which will be delivered back to the Garvan by the participant within 1 week of collection.
8. Visit 3:
    - Location: Garvan Institute and St Vincent's Hospital
    - Duration: 60 minutes
    - Detail: this visit is identical to visit 1 (except no repeat MRI). Participants will be greeted at the Garvan Institute. They will be escorted to their appointment for DXA scan at St Vincent's Clinic, then fibroscan and MRI scan at St Vincent's Hospital. They will be fitted with a CGM device at the Garvan Institute, provided their accelerometer instructions regarding preparation for visit 4A. They will collate a diet history using the Easy Diet App for a week prior to visit 4.
    - If necessary, instructions regarding insulin dosing, clamp preparation, use of diet diary and accelerometer may be provided over the phone, or via tele-conferencing
9. Visit 4A
    - Location: Garvan Institute
    - Duration: full day
    - Detail: this visit is identical to visit 2A.
        - At the conclusion of the clamp study, patients will be offered food and will be fitted with their second CGM device to use for 1 week.

- Participants will have the choice to stop the study drug after 1 week of CGM monitoring, or continue under the supervision of their GP or usual endocrinologist.

10. Visit 4B (optional)
    - Location: St Vincent's Hospital (Diabetes Centre)
    - Duration: 1-1.5 hours
    - Detail: this visit is identical to visit 2B.
11. Follow up Phone Call
    - Location: phone call
    - Duration: 5 minutes
    - Detail: A phone call will be made 1 week after participants stop the study drug to assess if insulin dose adjustment is required.
12. 3 month contact (post treatment cessation)
    - Location: phone call, stool and oral swab sample (home collection)
    - Duration: 5 minutes
    - Detail: 3 months after ceasing treatment, participants will provide a final stool sample and oral swab. Collection will be done at home, and participants will deliver the sample to the Garvan Institute within 1 week of collection.
13. Visit 5 (optional)
    - Location: Royal North Shore Hospital
    - Duration: 1 hour
    - Detail: beta hCG testing is performed to rule out pregnancy, then pQCT testing is completed.
        - Patients will indicate whether they consent to this visit during study enrolment.
        - However of those that opt-in to this visit, after data analysis, 8 normal participants, 8 T1D participants that are insulin resistant, and 8 T1D participants that are insulin sensitive will be invited to participate in this visit.

<u>Data Collection</u>:

- Data will be collected from multiple sources:
    - Results of outside pathology companies: including tests performed to satisfy inclusion and exclusion criteria. These tests will be requested and received by study investigator Dr Snaith.
    - Data obtained from physical measurements during a study visit
    - Results from investigations performed at the Garvan Institute and St Vincent's Hospital
    - CGM data: will be locally downloaded directly to a computer only available to study investigators.
    - Retinopathy assessment and SD-OCT, ERG and pupillometry reports will be collected by the orthoptist affiliated with the study, then analysed by ophthalmologists affiliated with the study.
    - MRI report: provided by AMRA Medical via St Vincent's Hospital radiology department
    - pQCT report: will be initially available to study investigators affiliated with Royal North Shore hospital, then shared with co-investigators
- Data will be recorded into excel spread sheets, or added to the secure REDCap data electronic spread sheet database.
- These spread sheets will be kept on a password-protected computer that is accessible only to staff involved in the study, and kept on the secure Garvan protected internal database.

<u>Outcome measures:</u>

There are numerous outcome measures of interest in this study:

- **Primary outcome measure:** hepatic insulin resistance (and change after metformin intervention) determined by insulin clamp study
- **Secondary outcomes**:
    - Peripheral insulin resistance (and change after metformin intervention)
    - anthropometrics (weight, waist circumference, blood pressure profile, determined by physical examination)
    - body composition (liver fat, total body fat, visceral fat, bone density determined by DXA scan)
    - hormones and metabolites (HbA1c, lipid profile, adiponectin, fasting glucose, c- peptide, non-esterified fatty acids, growth hormone, insulin-like growth factor 1, uric acid, sex hormone binding globulin, GDF15 - determined by blood tests)
    - indices of oxidative and inflammatory stress (c-reactive protein, VWF, e-selectin, t-PA, PAI, VCAM-1, ICAM-1) determined by blood test and radial artery applanation tonometry)
    - diabetes complications (albuminuria - determined by urine test, retinopathy status and retinal vascular changes)
    - insulin requirements, glycaemic variability, hypoglycaemia (determined by CGM and patient history)
    - safety measures (severe hypoglycaemia, time in hypoglycaemia, GI intolerance)
    - microbiome (oral and stool microbiome species, and metabolomics)
    - mitochondrial activity

These will be used to
1. Compare these parameters between T1D and participants without diabetes
2. Within T1D, if there is an association between the above parameters and insulin resistance patterns
3. Determine if these parameters predict response to metformin, or improve after treatment with metformin.

<u>Possible impacts of participants withdrawals:</u>
If participants withdraw, the study investigators may continue to recruit additional participants, to achieve the minimal number of participants to allow for adequate data analysis. This may prolong the period of data collection and duration of the study.

<u>Response to participant withdrawal:</u>
- The participant is free to withdraw from the research study at any time without prejudice.
- If the participant has been taking the study medication, they will be provided information regarding safe cessation of the drug. They will also be provided instructions on who to contact if there are any questions or concerns that arise after completing the study.
- If data has already been collected, the participant will be asked if they consent to the continued use of data by the investigators.

4.3. STUDY PROCEDURE RISKS

<u>Possible risks</u>
Related to study procedures:
- Pain or bruising caused from cannula insertion, or venepuncture

- Radiation exposure from DXA scanning, and pQCT
- Distress from fasting for 10 hours
- Calorimetry should not provide any risks, but in the event of claustrophobia, the mask will be immediately removed.
- Risk of hypoglycaemia during clamp study is minimised by close glucose monitoring, and will be treated immediately with an intravenous dextrose infusion.
- Risk of claustrophobia during MRI scan

Related to study medication (metformin):

- Common: gut intolerance causing bloating, nausea or loose bowel motions (these are usually short-lived and the risk reduced by starting with a low dose of medication)
- Possible: increased frequency of hypoglycaemia
- Uncommon: see list below

<u>Detailed information regarding metformin:</u> resourced from MIMs online (11)

- Approved name: Metformin XR
- Possible trade names: Diabex XR, Diaformin XR, Metex XR
- Manufacturer: Alphapharm
- Supplier of drug/device: Pharmacy
- Approved therapeutic indication, dosage/duration in Australia:
  - Approved for type 2 diabetes, polycystic ovarian syndrome
  - Duration of treatment: years
- Mode of action:
  - biguanide with antihyperglycaemic effects by lowering basal and postprandial plasma glucose levels. This is via 2 main mechanisms.
    1. at the liver: by reducing of hepatic glucose production: by inhibiting gluconeogenesis and glycogenolysis
    2. at the muscle: by increasing insulin sensitivity, improving peripheral glucose uptake and utilisation
  - Other actions: Metformin stimulates intracellular glycogen synthesis by acting on glycogen synthase, increases the transport capacity of all types of membrane glucose transporters (GLUT). In humans, independently of its action on glycaemia, metformin has favourable effects on lipid metabolism. This has been shown at therapeutic doses in controlled, medium-term or long-term clinical studies: metformin reduces total cholesterol, LDL cholesterol and triglyceride levels.
- Dosage regimen:
  - For this study: maximum tolerated dose up to 1.5g daily
  - Conventionally maximum dose: 3g daily
  - Conventional dosing: initially 500mg 1-3 times daily then increased according to response
- Mode of excretion: renal excretion
- Known adverse events:
  - Common (> 1%): nausea, vomiting, anorexia, diarrhoea
  - Infrequent (0.1-1%): rash
  - Rare (< 0.1%): lactic acidosis
- Known contra-indications or warnings:
  - Renal impairment or renal dysfunction
  - Use during time of metabolic acidosis (such as lactic acidosis or diabetic ketoacidosis)
  - Use during acute conditions that alter renal function (such as dehydration, severe infection, shock, intravascular iodinated contrast material)

- Use in acute or chronic disease that can cause tissue hypoxia (cardiac failure, recent myocardial infarction, respiratory failure, gangrene, shock, significant acute blood loss, pulmonary embolism, sepsis, pancreatitis)
- Elective major surgery
- Severe hepatic insufficiency, acute alcohol intoxication
- Warnings: lactic acidosis is a rare but serious metabolic complication of metformin accumulation. Reports of lactic acidosis have occurred in patients with significant renal failure (impairing clearance of metformin). The risk of lactic acidosis is reduced by avoiding prescribing metformin in the conditions listed above.

The use of metformin with insulin in type 1 diabetes has participated in previous clinical trials, and is common off-label practice.

The TGA CTN reference for our study is CT-2019-CTN-02001-1 v1.

Dispensing of study drug:

Participants will be randomised to study drug vs placebo after enrolment. The study drug (active and placebo) will be obtained from PCI compounding pharmacy (Melbourne, Victoria). This will be delivered to St Vincent's Hospital Pharmacy who will assist with randomisation and dispensing. The tablets will be dispensed as 500mg metformin XR tablets. Participants will be instructed to start with 1 tablet, then if tolerated after 1 week to increased to 2 tablets. Then if tolerated over 2 weeks to increase to 3 tablets daily then to remain on that dose.

4.4. RECRUITMENT AND SCREENING

Participants will be recruited over a 6 to 18 month period, beginning mid 2019 (once ethics has been secured).

Potential participants will be identified from several possible locations including:

- St Vincent's Hospital Type 1 diabetes clinics
- Westmead Hospital Type 1 diabetes clinics
- Private consulting rooms of Endocrinologists
- Through advertisement on notice boards displayed around the hospital precinct, including posters in outpatient areas or hospital foyers.
- Through advertisement on social media platforms

An information letter will be provided to clinicians that service clinics and private consulting rooms providing details of the study (see appendix). If the participant is interested in being screened for the study, then they may email the study coordinator email address (INTIMET@garvan.org.au).

During screening (phone or email):

- The potential participant will be briefed regarding the rationale for the study, and brief study procedure (see appendix Phone Script_Screening Questionnaire_INTIMET)
- Brief history to check relevant inclusion and exclusion criteria
    - Criteria that is confirmed on history:
        - Age

  - Date of diagnosis of diabetes (or confirming no known diabetes if not T1D)
  - Smoking status
  - Medications that affect glucose metabolism (glucocorticoids, antipsychotics, immunosuppressants).
  - Exposure to metformin
  - Alcohol intake
  - Weight change in the last 3 months
  - Pregnancy, breastfeeding, or plans for pregnancy over the duration of study
  - Major organ dysfunction (renal, hepatic, recent cardiac event, thyroid dysfunction)
  - Recent DKA
  - Recent severe hypoglycaemia requiring assistance
- If no clear exclusion criterion are met, then the study procedure, and risks will be discussed in further detail and the participant will be emailed the study PICF and the screening blood test request form. If participants have performed the relevant results recently, then we will seek permission to retrieve these results rather than duplicate the tests.
- Participants will be contacted with the results of screening blood tests to advise if they have met inclusion criteria. They will be invited to discuss any further consent queries during the first face-to-face visit.
- See study visits for further detail (section 5.2).

4.5. INFORMED CONSENT PROCESS

During the Screening/Enrolment visit, the study Investigator will explain the study aims, procedures, involvement, requirements and potential risks, and the informed consent will be signed before investigations are performed.

Our consent process highlights the participating in the study is voluntary, and that participants are able to withdraw from the study at any stage. Participants will receive a copy of the signed consent form. The original consent form will be kept in folder locked in a filing cabinet at the Garvan.

The consent document (see supplementary document) will differ for participants with diabetes compared to those without, as participants with diabetes will participate in an intervention phase of the project.

4.6. ENROLMENT PROCEDURE

Volunteers interested in participating in the study will contact the study team by phone or email.

Participants will be enrolled into the study after they pass the stage of phone questionnaire screening, screening blood tests, the informed consent process has been completed and if they have satisfied all inclusion and exclusion criteria.

The participant will be assigned a study enrolment number/ code.

4.7. RANDOMISATION PROCEDURE

Participants will be randomised using the minimisation program for allocation of patients to parallel groups, modified from Saghaei et al (10). Participants will be randomised to placebo or metformin using minimisation (Minitable program) modified from Saghaei et al (10). Factors were weighted for importance and included BMI (40% weighting), HbA1c (20% weighting), gender (20% weighting), age (20% weighting).
This will be supervised by an investigator not involved with recruitment or clinical care. Allocations will be communicated directly to St Vincent's Hospital clinical trials pharmacy.

5. TISSUE COLLECTION/BIOBANKING

This study will involve the collection of:

- Blood – 160mL (including plasma, serum and cell pellet)
- urine
- stool
- oral swabs

The collection of blood and urine is mandatory, but the collection of stool and oral swabs is optional.

Some tests are components of routine care including:

- urine ACR (albumin:creatinine ratio)
- blood tests – HbA1c, fasting glucose level, lipid profile, blood counts, liver and kidney tests

Other tests are for research purposes only including:

- stool and oral swab sample – for assessment of gut microbiome. Samples will be stored for future analysis.
- blood tests (essential for the study)
  - including adiponectin, c-reactive protein, VCAM-1, ICAM-1, uric acid level, HbA1c, lipid profile, adiponectin, fasting insulin, glucose, c- peptide, non-esterified fatty acids, free fatty acids, growth hormone, insulin-like growth factor 1, uric acid, sex hormone binding globulin, c-reactive protein, VWF, e-selectin, t-PA, PAI, VCAM-1, ICAM-1, GDF15
  - These tests are useful for assessment of primary and secondary outcomes, specifically to determine insulin clamp derived insulin resistance (primary objective), and to characterise metabolic and endothelial health status in each participant (secondary objective).

Samples will be coded so that results are not identifiable. They are re-identifiable, however strict measures will be taken to ensure participant privacy. These include limiting access of the study participant codes to only one investigator, who will keep the codes on a single password protected computer. No tissue in isolation will be identifiable, and will all be stored on-site. There will be no external storage of tissue. Blood and stool may be analysed offsite but not stored off-site. Offsite analysis will be performed by collaborators with our research group or by commercial laboratories (Laverty Pathology, Pathology West/ ICPMR Westmead Hospital). Their involvement in analysis is planned and necessary since we lack the resources to perform specialised analysis locally at the Garvan Institute. These collaborators may also require access to study related data. This is necessary to interpret of results in preparation for publication. All data will remain deidentified. Examples of offsite analysis sites include the Microbiome Research Centre (UNSW), Deakin University, University of Sydney/

NHMRC research centre, the University of Adelaide/ South Australian Health and Medical Research Institute. Samples sent off site are not identifiable.

Blood, oral swabs and stool will be kept and stored for up to 15 years after the completion of the project, and will then be destroyed by following Garvan local protocols. Urine will not be stored.

Stool and oral swabs will be stored for future analysis. Analysis of microbiome and metabolome is beyond the immediate goals of the project, but may be a topic of future interest.

There is no contemplated use of any tissue for commercial purposes, or for establishment of a tissue bank.

6.SAFETY

Adverse event

An adverse event for medicines is also referred to as any untoward medical occurrence in a patient or clinical investigation participant administered a pharmaceutical product and which does not necessarily have a causal relationship with this treatment.

An adverse reaction is any untoward or unintended response to an investigational medicinal product related to any dose administered.

A major adverse event may be any medical occurrence that results in: death, is life-threatening, requires hospital admission or prolongation of existing hospitalisation, persistent or significant disability or condition requiring medical or surgical intervention.

Adverse events related to this study

Minimal adverse events are expected from this study. Any conditions that place a participant at risk of an adverse event has been listed as an exclusion criterion.

Events that are expected as part of this study include:

- Minor bruising at the site of cannula insertion on the days of insulin clamp studies, or from the insertion of the continuous glucose monitor.
- Mild gut intolerance (loose bowel motion, nausea or bloating) after first starting the study medication (metformin). This is a commonly accepted side effect of metformin, and is reduced by starting the participant on a low dose of medication, then gradually increasing the dose.
- Possible hypoglycaemia. If metformin is an effective insulin sensitiser, the participant may require lower than usual insulin doses. Participants will be advised that their insulin doses may require adjustment and that advice can be provided either by study investigator Dr Snaith (an Endocrinologist), or the patient's own physician. Low glucose events can be a common occurrence in type 1 diabetes, and are rarely dangerous. However the study medication may cause extra episodes.

Possible adverse events are further detailed in section 5.3 (study procedure risks).

Safety will be monitored throughout the study by:

- Checking tolerance of study medication and any adverse events at all study visits, and with a follow up phone call 2 weeks after initially starting the study drug.

- Providing participants with contact details of the study investigators to provide easy avenues for discussion of any concerns as they arise.
- Informing the participant's usual physician of their participation in the study.

6.1. Adverse Event Reporting

Adverse event reporting will meet the requirements of the NHMRC Position Statement 'Safety monitoring and reporting in clinical trials involving therapeutic goods' (2016) (12).

6.2. Serious Adverse Event Reporting

Serious adverse event (SAE):
For medicines, also referred to as serious adverse drug reaction, any untoward medical occurrence that at any dose:

- results in death;
- is life-threatening;
- requires in-patient hospitalisation or prolongation of existing hospitalisation;
- results in persistent or significant disability/incapacity;
- is a congenital anomaly/birth defect; or
- is a medically important event or reaction.

NOTE: The term 'life-threatening' in the definition of 'serious' refers to an event in which the patient was at risk of death at the time of the event; it does not refer to an event/reaction that hypothetically might have caused death if it were more severe.

Any serious adverse events will be reported in accordance with the NHMRC Position Statement 'Safety monitoring and reporting in clinical trials involving therapeutic goods' (12).

6.3. Data Safety and Monitoring Board

This is an investigator initiated clinical trial. Any significant adverse events will be reported directly to the HREC, according to Garvan Institute policies and practices.

6.4. Early Termination

It is not anticipated that there will be any likely circumstance triggering early termination of the study in totality as the trial medication is considered safe in the study population and the risk of adverse event is rare.

As the medication trialed in the present study is safe in similar cohorts, we do not expect any circumstances that will require termination of the study.

The study Investigators will follow the guidelines detailed in the National Statement on Ethical Conduct in Human Research (NHMRC, 2007) if the project is to be discontinued before the expected date of completion.

7. BLINDING AND UNBLINDING

This is a double-blinded study. We will blind both participants and investigators to whether participants with T1D have received metformin or placebo.

Whether the participant has T1D will be known to the investigators performing data collection and study visits, as T1D participants will have additional CGM, will progress to the second

stage of the study that involves testing a therapy, and requires additional monitoring and altered protocols during the clamp study.

8. OUTCOMES AND FUTURE PLANS

The results of urine pregnancy tests will be made available immediately. The results of routine tests will be made available to the patient and their usual clinician unless they specify that they wish these results not to be known. These results will be made available, and printed on request after initial pre-enrolment blood tests.

The results of the project will be published as journal articles, and will be submitted for Dr Jennifer Snaith's PhD thesis. The outcomes of research will be summarised into language understandable to a non-academic audience, which will be sent as a letter to all research participants.

Project closure

At the completion of the project, a closure report will be provided. This will be done once all research activities are completed and when all contact with participants has been finalised.

The study close out procedure includes:

1. Data – confirming that all data collection is complete and entered into the database.
2. Adverse events – ensuring that all adverse event and serious events have been reported and reconciled according to protocol reporting requirements.
3. Consent – confirm that signed consent forms are on file for all participants.
4. Specimens – ensure that all specimens have been tracked, logged, processed or stored for future use if required. Ensure that specimens collected for future use have been adequately processed, labelled, coded and stored. Confirm site processes for identification and disposition of future use specimens connected to participants who withdraw consent or do not consent for their samples to be saved. Confirm destruction per institutional policies of specimens not identified for future analysis.
5. Analysis and manuscripts – complete data analysis, and finalise manuscripts.

Regular annual progress reports and a final report will be submitted using forms provided through the REGIS system.

Future use of data or follow up research

The option for use of data for related or follow up research will be included in the consent form.

Possible future research may include but is not limited to linking insulin resistance measures and metabolic/ vascular status data with gut microbiota patterns. Although there are plans to collect and store stool, the scope of the current project does not currently include stool analysis. If funding is secured, and there is ongoing interest into researching the gut microbiome, the stool samples may be studied in future. There are also future plans to examine the effect of metformin on mitochondrial health. Cell pellet will be stored then analysed in future pending acquisition of funding.

Any future research and use of data will be in the area of type 1 diabetes research.

If there is any additional tissue or data that is required to extend the current topic of research, a supplementary HREC submission will be made.

9.STATISTICAL CONSIDERATIONS

Sample size or power calculation

The study's target sample size was derived from a power calculation using reported data from a study of metformin use in adolescent T1D (13). This is the only published study that has examined change in hepatic insulin resistance in T1D following treatment with metformin. A sample size of 20 treated with metformin, and 20 with placebo is expected to provide results with alpha error probability of 0.05 and 87% power.

Other studies in similar populations have participant numbers similar to our trial design including the Bjornstad et al and Sarnblad et al studies which included 24 and 16 participants metformin treated T1D participants (14,15). The demands of rigorous insulin clamp protocols limit the number of participants that can be processed.

Statistical Plan

Similar studies have reported up to a 20% participant drop out rate.

Differences in characteristics between non-diabetic and T1D as a group will be determined using Mann-Whitney and t-tests. Linear regression analyses will be used to investigate the possible association between the level of insulin resistance at each site (liver–EGP, muscle–GIR) and individual baseline characteristics. Similarly, the same techniques will be used to assess possible associations between the change in insulin resistance at each site (liver – change in EGP, muscle – change in GIR) after metformin treatment. Hence possible predictors of response will be identified. Generalised linear mixed modelling will be used to analysis primary and secondary outcome measures to determine changes after metformin treatment, per main statistical analysis plan.

Confounding factors and potential bias

Currently, insulin resistance is reliably determined with clamp measurements only, and is not reliably predicted by clinical features. With this in mind, we will not specifically select for T1D participants in which increased insulin resistance is suspected (eg. elevated BMI, high insulin requirements). There may be volunteer bias in both T1DM and non-diabetic groups skewing towards more fit and health literate individuals, but the bias would be shared between both groups. Age and BMI will be balanced in participants and controls to reduce cofounding from those factors.

10.CONFIDENTIALITY AND STORAGE AND ARCHIVING OF STUDY DOCUMENTS

The Garvan Institute has internal policies regarding confidentiality and storage of research documents (16). UNSW Research Code of Conduct Principles will also be followed (17).

Stored data will be in the form of:

- Electronic
  - Word documents, excel documents, CGM download reports
  - Electronic data will be stored on a single password protected computer at the Garvan Institute.
  - Secure electronic databases (eg. REDcap)

- Paper
    - Eg. consent forms

Each participant will be associated with an individual program-generated code that will be used to identify their study documents, data and specimen collected during the study. The re-identifiable code will be documented in the participant's record and on all study documents. All participants' data will be stored on a paper hard copy, in electronic format on a computer file on the Garvan server and using the REDcap database platform (https://www.project-redcap.org/).

Some data relating to the randomization will be stored on Garvan servers. Access will be granted to limited, essential personnel in a de-identified form. Paper hard copies will be kept in a locked cabinet and electronic files on a password protected folder with access granted to the study team. Re-identifiable blood, stool, plasma and serum samples will be kept at the Garvan Institute's specialised storage facilities. The Garvan Institute is also a secure location, with sign in processes and is not accessible by the general public.

The images from MRI scans will be deidentified, and a study code used. The images are then sent to the software company (AMRA medical) for analysis. Images are sent using secure cloud-based software. AMRA medical will have no knowledge of the identity of the individual. As per their licencing agreement, AMRA may retain the deidentified data for internal or external use (including publication) on the proviso that it is there is no possibility of identification of the individual.

The CGM data will be de-identified and a study code used. Data extraction requires import of data from the CGM receiver to a commercial application using cloud-based servers. Dexcom may access and retain this data but Dexcom will have no knowledge of the identity of the individual, and the data will not be identifiable.

In accordance with the NHMRC Code for Responsible Conduct of Research, data will be stored for up to 15 years from the date of publication (18).

When the specified period of retention has lapsed, data will be disposed of in a secure and safe manner in accordance with UNSW Record Keeping Policy (19).

11.OTHER STUDY DOCUMENTS

Document
INTIMET_Study Protocol
PICF Participants T1D_INTIMET
PICF Participants without DM_INTIMET
Advertisement with tabs _INTIMET
Advertisement_INTIMET
Clamp Study Prep Letter_INTIMET
Data Collection_Clamp Worksheet_INTIMET
Data Collection_History and Physical Examination Form_INTIMET
Food Diary_INTIMET
Letter Enrolment bloods_INTIMET
Letter Invitation to Physicians_INTIMET
Letter Invitation_Participants T1D_General_INTIMET
Letter Invitation_Participants without DM_General_INTIMET
Letter Notification to Physicians T1DM_INTIMET
Letter Preenrolment T1D_INTIMET
Phone Script_Screening Questions

12.RESOURCES

Secured funding includes a $150 000 Millennium Award Grant from Diabetes Australia. The funds will be available over 2 years, and is available after February 2019. A further $50 000 has been awarded by the St Vincent's Clinic Foundation 2020 Tancred Award.

The resources necessary for the study to be conducted include:

- Personnel: research nurse
- Facilities: investigation room (available at the Garvan)
- Equipment:
    - DXA machine
    - Fibroscan machine
    - MRI machine
    - Applanation tonometry machine
    - Computer and software
- Consumables:
    - Blood tubes and syringes
    - Urine container
    - Urine pregnancy test kit
    - Stool collection kit (OMNIgene.GUT), and oral swab collection kit
    - Intravenous cannulae, venepuncture equipment, intravenous tubing

- Intravenous fluid and giving set
- Supply of insulin
- Deuterated glucose
- Prepacked meal to provide participants after clamp study
- Metformin supply
- Continuous glucose monitor (Medtronic iPro and enlite sensors, Dexcom G6)

13.REFERENCES

1. Data Snapshots [Internet]. [cited 2018 Nov 25]. Available from: https://www.ndss.com.au/data-snapshots

2. Greenfield JR, Samaras K, Chisholm DJ. Insulin Resistance, Intra-Abdominal Fat, Cardiovascular Risk Factors, and Androgens in Healthy Young Women with Type 1 Diabetes Mellitus. J Clin Endocrinol Metab. 2002;87(3):1036–40.

3. Schauer IE, Snell-Bergeon JK, Bergman BC, Maahs DM, Kretowski A, Eckel RH, et al. Insulin Resistance, Defective Insulin-Mediated Fatty Acid Suppression, and Coronary Artery Calcification in Subjects With and Without Type 1 Diabetes. Diabetes. 2011 Jan;60(1):306–14.

4. Wiernsperger NF, Bailey CJ. The antihyperglycaemic effect of metformin: therapeutic and cellular mechanisms. Drugs. 1999;58 Suppl 1:31–9; discussion 75-82.

5. Effect of intensive blood-glucose control with metformin on complications in overweight patients with type 2 diabetes (UKPDS 34). The Lancet. 1998 Sep;352(9131):854–65.

6. Chen DL, Liess C, Poljak A, Xu A, Zhang J, Thoma C, et al. Phenotypic Characterization of Insulin-Resistant and Insulin-Sensitive Obesity. The Journal of Clinical Endocrinology & Metabolism. 2015 Nov;100(11):4082–91.

7. Finegood DT, Bergman RN, Vranic M. Estimation of Endogenous Glucose Production During Hyperinsulinemic-Euglycemic Glucose Clamps: Comparison of Unlabeled and Labeled Exogenous Glucose Infusates. Diabetes. 1987 Aug 1;36(8):914.

8. Nürnberger J, Keflioglu-Scheiber A, Opazo Saez AM, Wenzel RR, Philipp T, Schäfers RF. Augmentation index is associated with cardiovascular risk. J Hypertens. 2002 Dec;20(12):2407–14.

9. Greenfield JR, Samaras K, Hayward CS, Chisholm DJ, Campbell LV. Beneficial Postprandial Effect of a Small Amount of Alcohol on Diabetes and Cardiovascular Risk Factors: Modification by Insulin Resistance. J Clin Endocrinol Metab. 2005 Feb 1;90(2):661–72.

10. Saghaei M, Saghaei S. Implementation of an open-source customizable minimization program for allocation of patients to parallel groups in clinical trials. Journal of Biomedical Science and Engineering. 2011;04(11):734–9.

11. Full Product Information [Internet]. [cited 2019 Jan 11]. Available from: https://www.mimsonline.com.au.acs.hcn.com.au/Search/FullPI.aspx?ModuleName=Product%20Info&searchKeyword=metformin&PreviousPage=~/Search/QuickSearch.aspx&SearchType=&ID=88690001_2

12. Safety monitoring and reporting in clinical trials involving therapeutic goods | NHMRC [Internet]. [cited 2019 Jan 11]. Available from: https://nhmrc.gov.au/about-us/publications/safety-monitoring-and-reporting-clinical-trials-involving-therapeutic-goods

13. Cree-Green M, Bergman BC, Cengiz E, Fox LA, Hannon TS, Miller K, et al. Metformin Improves Peripheral Insulin Sensitivity in Youth with Type 1 Diabetes. The Journal of Clinical Endocrinology & Metabolism [Internet]. 2019 Apr 2 [cited 2019 Jun 11];

Available from: https://academic.oup.com/jcem/advance-article/doi/10.1210/jc.2019-00129/5423566

14. Bjornstad P, Schäfer M, Truong U, Cree-Green M, Pyle L, Baumgartner A, et al. Metformin Improves Insulin Sensitivity and Vascular Health in Youth With Type 1 Diabetes Mellitus: Randomized Controlled Trial. Circulation. 2018 Dec 18;138(25):2895–907.

15. Sarnblad S, Kroon M, Aman J. Metformin as additional therapy in adolescents with poorly controlled type 1 diabetes: randomised placebo-controlled trial with aspects on insulin sensitivity. Eur J Endocrinol. 2003 Oct 1;149(4):323–9.

16. garvan-privacy-policy-may13.pdf [Internet]. [cited 2019 Jan 13]. Available from: https://www.garvan.org.au/about-us/policies/garvan-privacy-policy-may13.pdf

17. Data Handling Guidelines | Data Governance [Internet]. [cited 2019 Feb 5]. Available from: https://www.datagovernance.unsw.edu.au/node/16

18. National Statement on Ethical Conduct in Human Research (2007) - Updated 2018 | NHMRC [Internet]. [cited 2019 Feb 5]. Available from: https://nhmrc.gov.au/about-us/publications/national-statement-ethical-conduct-human-research-2007-updated-2018#block-views-block-file-attachments-content-block-1

19. Document Control and Recordkeeping at UNSW | Health & Safety [Internet]. [cited 2019 Feb 5]. Available from: https://safety.unsw.edu.au/office-safety-toolkit/document-control-and-recordkeeping-unsw

Statistical Analysis Plan for the INTIMET study (Insulin Resistance in Type 1 Diabetes Managed with Metformin)

Abstract

This document provides the statistical analysis plan (SAP) for the INTIMET study (Insulin Resistance in Type 1 Diabetes Managed with Metformin), a randomised double-blinded placebo-controlled trial, designed to evaluate the effect of metformin on insulin resistance and cardiometabolic health in type 1 diabetes. This trial was prospectively registered within the Australian and New Zealand Clinical Trials Registry (ACTRN12619001440112). The study protocol has previously been published (Snaith JR et al, Diabetic Medicine 2021).

Table of Contents

1. Section 1: Administrative information *3*

1.1. **Title and trial registration** 3

1.2. **SAP version** 3

1.3. **Protocol version** 3

1.4. **SAP REVISION HISTORY** 3

1.5. **Roles and responsibilities** 4

2. Section 2: Introduction *4*

2.1. **Background and rationale** 4

2.2. **Aims and objectives** 5

3. Section 3: study methods *5*

3.1. **Trial design** 5

3.2. **Randomisation** 6

3.3. **Sample size** 6

3.4. **Framework** 6

3.5. **Statistical interim analysis and stopping guidance** 6

3.6. **Timing of final analysis** 7

3.7. **Timing of outcome assessments** 7

4. Statistical principles *7*

4.1. **Confidence intervals and p-values** 7

4.2 Adherence and protocol deviations 7

4.3 Analysis populations 7

5. Section 5: Trial population *7*

5.1. **Screening data** 7

5.2. **Eligibility** 8

5.3. **Recruitment and withdrawal/ follow up** 8

5.4. **Baseline patient characteristics** 8

6. Analysis *10*
6.1. Outcome definitions **10**
6.2. Analysis methods **11**
6.3. Missing data **12**
6.4. Additional analysis **13**
6.5. Harms **13**
6.6. Statistical software **13**
7. Other *13*
7.1. References **13**

1. SECTION 1: ADMINISTRATIVE INFORMATION

1.1. TITLE AND TRIAL REGISTRATION

The INTIMET study (Insulin Resistance in Type 1 Diabetes Managed with Metformin) is a parallel group, superiority, randomised double-blind placebo-controlled trial examining whether metformin is superior to placebo to reduce insulin resistance and improve cardiometabolic parameters in type 1 diabetes. INTIMET includes a cross-sectional study of both type 1 diabetes with age- BMI and gender-matched controls without diabetes to enable a detailed study of the phenotype of insulin resistance in type 1 diabetes.

Trial registration:
Australian New Zealand Clinical Trials Registry (ANZCTR) ACTRN12619001440112
First registered: 17/10/2019
Universal trial number: U1111-1238-8090

1.2. SAP VERSION

SAP version 3.0, Dated 24/3/23. This SAP version follows guidelines for SAP reporting (Gamble et al. JAMA 2017).[1]

1.3. PROTOCOL VERSION

This SAP is based on the original protocol approved by the St Vincent's Hospital Human Health Research Ethics Committee, approved May 2019 (2019/ETH00379) and the published study protocol (https://doi.org/10.1111/dme.14564).[2]

1.4. SAP REVISION HISTORY

Original version: published study protocol, submitted 14/12/2020, accepted 24/3/2021

Justification of revisions:

Relevant SAP section	Change made	Reason
1.5 – roles and responsibilities	Change in study statistician	Change in UNSW staffing
4 – statistical principles	Increased detail on specifics of statistical plan	Original SAP did not provide full level of detail.
6.1 – outcome definitions	Revising low-dose EGP as the primary endpoint	The measure of low-dose EGP is more robust than basal Ra as a measure of hepatic insulin sensitivity in type 1 diabetes, due to the influence of the insulin infusion protocol during the basal stage of the clamp (ie. variable infusion to achieve euglycaemia impacting basal Ra measures).

	Change from ANCOVA to generalised linear mixed modelling (GLMM) as the primary analysis method, with the primary endpoint assessed by the time-by-treatment interaction.	Advice following change in statistician that GLMM provides more precise parameter estimates than ANCOVA and aligns the primary and secondary analysis methods (previously GLMM was proposed only for secondary analyses).

1.5. ROLES AND RESPONSIBILITIES

Names, affiliations, and roles of SAP contributors:

Prof Jerry Greenfield, MBBS (Hons), PhD, FRACP
Garvan Institute of Medical Research, University of New South Wales, Sydney, Australia, St Vincent's Hospital Sydney
Responsibility: Chief Investigator

Dr Jennifer Snaith, MBBS(Hons), BMedSc, FRACP
Garvan Institute of Medical Research, University of New South Wales, Sydney, Australia, St Vincent's Hospital Sydney
Responsibility: Preparation of SAP, study coordinator, performed all studies and data collection

Dr Nick Olsen, BSci (Hons), PhD
Mark Wainwright Analytical Centre, University of New South Wales, Sydney, Australia
Responsibility: Lead Statistician

2.SECTION 2: INTRODUCTION

2.1. BACKGROUND AND RATIONALE

Type 1 diabetes affects 120 000 individuals in Australia and the incidence is rising.[3] It is a condition typified by autoimmune destruction of pancreatic beta cells leading to insulin deficiency necessitating insulin replacement. People with type 1 diabetes have an increased cardiovascular (CV) risk and excess mortality relative to general population. However, this increased risk is not explained by hyperglycaemia alone.

Insulin resistance is under recognised in type 1 diabetes and is relevant to altered metabolic pathways in fat, liver, muscle and other tissues. The Garvan laboratory has previously established that type 1 diabetes individuals are more insulin resistant than their non-diabetic peers. This was determined using insulin clamp studies, the gold standard measure of insulin resistance.[4] However, there remain key unanswered questions with regards to insulin resistance in type 1 diabetes; its cause, its consequences and its phenotype, including whether it arises from liver, muscle or both. Heterogeneity in the degree and tissue site of insulin resistance may explain diversity in metabolic phenotypes in type 1 diabetes, where some individuals are predisposed to metabolic syndrome with weight gain, increased insulin requirements, adverse lipid and blood pressure profiles and increased cardiovascular risk. This is also strongly suggested by the finding that type 1 diabetes patients with greater clamp-derived insulin

resistance associate with greater degrees of coronary artery calcification.[5] Heterogeneity in insulin resistance may also explain findings of variable responses to metformin in type 1 diabetes.

Metformin is an anti-hyperglycaemic agent that improves glycaemia in type 2 diabetes by lowering hepatic glucose output, and increasing peripheral glucose uptake in muscle.[6] It is thought to be beneficial to CV outcomes in type 2 diabetes.[5] Metformin has potential for repurpose in type 1 diabetes. Metformin studies in type 1 diabetes have not demonstrated consistent results, and at most have shown only minor improvements in weight, cholesterol, glycaemia and without a clear reduction in CV risk.[7] Although insulin replacement has been the mainstay of treatment in type 1 diabetes, there may be rationale for adjunctive agents to address cardiometabolic risk and may reduce insulin resistance.

Study rationale:
The role and pathogenesis of insulin resistance in type 1 diabetes is not well understood. It has been established that type 1 diabetes individuals are more insulin resistant than individuals without diabetes, but the tissue site of insulin resistance (muscle vs liver vs both) is not well characterised. Furthermore, the phenotype of insulin resistance, and whether liver vs muscle insulin resistance has consequences for lipid and blood pressure profiles, weight, or cardiovascular risk, and if remediable with therapy requires further study.

Our trial is the first study to examine tissue specific patterns of insulin resistance in adults with type 1 diabetes and the effect of metformin on tissue specific insulin resistance.

This study has the potential to change treatment paradigms with an increased focus on treatment of insulin resistance in type 1 diabetes, and to define whether tissue specific insulin resistance is a candidate target for adjunctive therapy.

2.2. AIMS AND OBJECTIVES

Hypotheses:
1) In type 1 diabetes, insulin resistance aligns with worse metabolic and cardiovascular profiles (higher blood pressure, adverse lipid profile, visceral and hepatic steatosis).
2) Treatment with metformin will improve hepatic insulin sensitivity in T1D relative to placebo.

The overall aims of this project are to:
1. Characterise patterns of insulin resistance in muscle and liver in type 1 diabetes and determine the impact on metabolic phenotype.
2. Investigate whether metformin improves insulin resistance in type 1 diabetes and to determine the predictors of response to metformin in type 1 diabetes.

3.SECTION 3: STUDY METHODS

3.1. TRIAL DESIGN

The INTIMET study has a 2-part design:

Part 1: Cross-sectional study of 40 adults with type 1 diabetes and 20 adults as a non-diabetes comparator group (2:1 ratio) to extensively characterise the phenotype of insulin resistance in adults with type 1 diabetes.

Part 2 (main study): Double blinded randomised placebo-controlled trial of 6 months of metformin therapy (1.5g XR daily) in adults with type 1 diabetes (1:1 randomisation).

The study design has been developed in accordance with:

- SPIRIT statement for interventional trials: http://www.spirit-statement.org/
- CONSORT guidelines for randomised trials: http://www.consort-statement.org/

3.2. Randomisation

Randomisation of metformin to placebo will be 1:1 using minimisation (Minitable program) modified from Saghaei et al [8]. Factors will be weighted for importance and included BMI (40% weighting), HbA1c (20% weighting), gender (20% weighting), age (20% weighting). The factors will be provided to a clinician independent to the study who will then perform the randomisation and communicate the allocation directly to the St Vincent's Hospital clinical trials pharmacy.

Research staff (including those involved with recruitment and outcome assessors), participants, care providers and data analysts will remain blinded to the treatment allocation. The dispensing pharmacist will not have contact with study participants.

The central study coordinator will code the 2 treatment arms as Group A and Group B (determined by coin toss) before providing the data to the statistician.

3.3. Sample size

A sample size of 40 participants (20 per treatment arm) was determined to provide 80% power at a two-sided significance level of 0.05 to detect a mean difference of 0.3 mg/kg/min with a pooled standard deviation of 0.3 mg/kg/min (effect size of 1) in the primary outcome measure of Ra (a measure of endogenous glucose production; EGP) at week 26, assuming a 15% attrition rate. This effect size estimation was taken from Cree-Green et al, a study of the effect on EGP in an adolescent population with type 1 diabetes treated with metformin for 3 months.[9]

3.4. Framework

Primary and secondary outcomes will be assessed using a superiority framework, expecting that participants randomised to metformin will improve more than those randomised to placebo.

3.5. Statistical interim analysis and stopping guidance

No stopping rule is defined. Recruitment will continue until the target of n=40 participants with type 1 diabetes is reached. This is to ensure a power of 80% anticipating a potential 15% loss to follow-up.

3.6. TIMING OF FINAL ANALYSIS

Analysis will occur after the final volunteer completes their final study visit and all tracer clamp data (required for primary outcome data) is processed and available for interpretation.

3.7. TIMING OF OUTCOME ASSESSMENTS

Timing of outcome assessments per Table 3 of published protocol paper.[2]

4.STATISTICAL PRINCIPLES

4.1. CONFIDENCE INTERVALS AND P-VALUES

All assessments of between group effects will use two-sided tests with a 5% significance level, p=0.05. Confidence intervals will be presented as 95% and two-sided.

Regarding secondary outcome analysis, the number of statistically significant analysis that we expect from chance alone based on the number of subgroup analysis will be acknowledged in data interpretation. The primary outcome is well-defined (change in low-dose EGP from baseline to 26 weeks).

4.2 ADHERENCE AND PROTOCOL DEVIATIONS

Adherence to intervention will be assessed by tablet count upon final tablet return. Poor compliance will be defined as return of >25% of the target tablets for the max tolerated dose of that individual.

A protocol deviation will be defined as >15% loss to follow up or less than 40 volunteers with type 1 diabetes undergoing randomisation. Any protocol deviations will be reported in the final report.

4.3 ANALYSIS POPULATIONS

Analysis of all randomised subjects will be conducted using an intention-to-treat approach (ITT) according to the treatment they were allocated to receive. This will be the primary population for the analysis.

5.SECTION 5: TRIAL POPULATION

5.1. SCREENING DATA

The time frame for recruitment (start and end date), first and last participant enrolment and data collection will be reported. The total number of subjects screened for eligibility will be declared in the CONSORT diagram.

5.2. ELIGIBILITY

<u>Summary of eligibility criteria</u>
Inclusion:

- Age range: 20-55 years (and pre-menopausal if female)
- Type 1 diabetes:
    1. Disease duration > 10 years (ie. chronic exogenous insulin exposure)
    2. Insulin deficiency (fasting c-peptide < 0.3nmol/L)
    3. HbA1c less than or equal to 9.5%

Exclusion:

1. Current smoking
2. Current or planned prescription of medications that affect glucose metabolism (glucocorticoids, antipsychotics, immunosuppressants).
3. Exposure to metformin within the last 30 days
4. Alcohol intake > 20g/day in women or > 40g/day in men
5. Weight change > 5% in last 3 months or history of bariatric surgery
6. Pregnancy, breastfeeding, or childbearing potential not willing to avoid pregnancy during the study.
7. Known major organ dysfunction (eGFR < 60, liver disease transaminases > 3 times the upper limit of normal, cardiac event within the last 6 months, current cancer or uncontrolled thyroid dysfunction).
8. Diabetic ketoacidosis or severe hypoglycaemia (hypoglycaemia requiring third-party assistance) in the last 6 months.
9. A history of a psychological illness or condition that would interfere with the patient's ability to understand the requirements of the study.

5.3. RECRUITMENT AND WITHDRAWAL/ FOLLOW UP

The following information will be included in the CONSORT flow diagram:

- all volunteers assessed for eligibility
- all volunteers meeting exclusion criteria, and the reason for exclusion
- all volunteers eligible for inclusion
- all volunteers not consenting (and reasons)
- all patients randomised
- all patients that undergo assessment at 3 and 6 months
- withdrawals with reasons and timing and treatment arms
- patients included in the ITT, per protocol and as treated analyses in both treatment arms

5.4. BASELINE PATIENT CHARACTERISTICS

List of baseline characteristics to be summarised:

Age (years)

Sex (% male)
HbA1c (%, mmol/mol)
BMI (kg/m^2)
SBP (mmHg)
DBP (mmHg)
Diabetes duration (years)
Ethnicity (% Caucasian)
Family history ischaemic heart disease (n,%)
Family history type 2 diabetes (n,%)
Waist to hip ratio
Insulin delivery method
Pump (n, %)
MDI (n, %)
Total daily insulin dose (U/Day)
Time in range (%)
Time in hyperglycaemia (%)
Time in hypoglycaemia (%)
Glycaemic variability (CV)
Retinopathy grading (n,%)
Maculopathy grading (n,%)
Augmentation index (AIx)
CAP (dB/m)
MRI liver fat (%)
MRI muscle fat infiltration (%)
MRI abdominal subcutaneous adipose issue volume (L)
DXA visceral adipose tissue (g)
DXA total fat mass (kg)
DXA total fat free mass (kg)
Total cholesterol (mmol/L)
HDL (mmol/L)
LDL (mmol/L)
Triglycerides (mmol/L)
Bilirubin (µmol/L)
Total protein (g/L)
Albumin (g/L)
Total globulin (g/L)
ALT (U/L)
AST (U/L)
GGT (U/L)
ALP (U/L)
Uric acid (mmol/L)
IL-6 (pg/mL)
sICAM-1 (ng/mL)
SE-selectin (ng/mL)
Adiponectin (µg/mL)
IGF1 (nmol/L)
SHBG (nmol/L)
GDF-15 (pg/mL)

These will be presented as type 1 diabetes vs control for the cross-sectional study, and as metformin vs placebo for the main RCT.

The number of available measurements of the baseline characteristics will be reported.

6. ANALYSIS

6.1. OUTCOME DEFINITIONS

For detailed explanation of outcome definitions, refer to outcomes section of trial registration listing: https://www.anzctr.org.au/Trial/Registration/TrialReview.aspx?id=378197&isReview=true

In summary:
Primary endpoint: hepatic insulin sensitivity, assessed using EGP* during the low dose insulin phase of the hyperinsulinaemic-euglycaemic clamp (with deuterated glucose tracers).

*Note: The published protocol reports both basal Ra and low-dose EGP as a primary outcome measure. This statistical analysis plan reports a change in the definition of the primary outcome measure to low-dose EGP alone as a single primary outcome measure. The rationale for this change was due to concerns regarding the variable insulin infusion used during the basal stage of the clamp, impacting the validity of the basal Ra measure, whereas the low-dose EGP measure would not be impacted by clamp protocol factors. The change to the primary outcome was adopted after the protocol was published and registered in ANZCTR but before the final data lock and analysis.

Secondary endpoints:
Refer to table 1 per published protocol.[2]

**SECONDARY endpoints**	**Method of assessment**
Peripheral muscle insulin sensitivity	GIR during the high dose insulin phase of the hyperinsulinaemic-euglycaemic clamp
Adipose tissue insulin sensitivity	NEFA concentrations during the low dose insulin phase of the hyperinsulinaemic-euglycaemic clamp
Glycaemic control	HbA1c
Glucose variability<br>Glucose time in range (3.9-10mmol/L)<br>Time in hypoglycaemia (%)<br>Time in hyperglycaemia (%)<br>Glycaemic variability (CV)	Continuous glucose monitoring
Total daily insulin dose<br>Basal insulin dose<br>Bolus insulin dose	Review of participant logbook or insulin pump records
Weight/ BMI	Digital scales, stadiometer
Waist circumference, hip circumference	Tape measure
Blood pressure	Digital sphygmomanometer

Metabolic and vascular markers including:<br>Lipid profile (total, LDL, HDL cholesterol, triglycerides)<br>Adiponectin<br>Sex hormone binding globulin<br>C-reactive protein<br>GDF-15	Assays (serum or plasma)
Hepatic stiffness<br>Hepatic fat	Transient elastography (fibroscan), Liver MRI
Arterial stiffness (basal and insulin responsiveness)	Heart rate adjusted augmentation index assessed by radial artery applanation tonometry
Total fat mass<br>Fat-free mass<br>Visceral fat	Whole body DXA
Gastrointestinal microbiota	Stool analysis

Abbreviations: EGP, endogenous glucose production; GIR, glucose infusion rate; NEFA, non-esterified fatty acids; HbA1c, haemoglobin A1c; GDF-15, growth/differentiation factor-15; MRI, magnetic resonance imaging; DXA, dual-energy x-ray absorptiometry.

GIR and EGP data will be adjusted to fat free mass of the individual (determined from DXA scan).

Note: Indirect calorimetry was planned for, but data collection was aborted due to infection control issues during the COVID-19 pandemic.

6.2. ANALYSIS METHODS

Data distribution will be assessed by visual inspection of data histograms and quantile plots and supported by Shapiro-Wilk, skewness and kurtosis statistics. Normally distributed data will be presented as mean and standard deviation (SD) and analysed using parametric tests. Skewed data will be presented as median and interquartile range (IQR) then analysed with non-parametric tests or as log transformed data analysed with parametric tests. Categorical data will be presented as counts and percentages.

Effect estimates will include confidence intervals.

Primary outcome:
The primary analysis will compare intervention groups (metformin vs placebo) to assess whether there is a difference in hepatic insulin resistance (EGP during the low-dose phase of the hyperinsulinaemic-euglycaemic clamp; LD-EGP) after 26 weeks. The mean change in LD-EGP (delta EGP) from baseline to 26 weeks will be assessed using a generalised linear mixed model. These models will include a random intercept for each individual, with absolute LD-EGP as the response variable at each time point. The distribution and link will be determined empirically by residual versus fixed-effect prediction plots. Time, treatment group, and group by time interaction are pre-specified predictor variables. These same models will also be presented in a re-parameterised form with time, and group by time interaction (not including a main effect for treatment group), as group differences in the response variable at baseline can obscure the interpretation of the group by time interaction.[11]

The primary outcome for LD-EGP will be the treatment by time interaction.

We will also report the pre-post difference (with 95% confidence intervals) of the estimated marginal means with treatment group as the pairwise contrast.

We will systematically assess models as:

1. Unadjusted
2. Minimally adjusted using select physically plausible covariates (age, sex, baseline HbA1c and baseline BMI).

Secondary outcomes:
Secondary endpoints that relate to a change from baseline measurement will be analysed as per primary endpoint analyses.

Correlations between change in insulin resistance and change in cardiometabolic factors or laboratory measurements will be analysed. We will explore the association between metabolic parameters and the response to metformin intervention.

Assumptions for models will be checked. Regression models will be checked by visually inspecting residual plots for evidence of heteroscedasticity and linearity. Other model diagnostics such as the variance inflation factor (VIF) for collinearity will be checked.

Type 1 diabetes vs control analysis:
Two sample tests and Chi-square or fisher's exact tests will be used to compare baseline characteristics between volunteers with and without type 1 diabetes.

Correlations will be performed using Pearson or Spearman's correlations according to the normality of the data, or partial correlation if controlling for variables, to determine relationships between insulin sensitivity and cardiometabolic variables.

6.3. MISSING DATA

The nature of missing data relevant to the primary endpoint will be determined to be missing at random (MAR), missing completely at random (MCAR) or missing not at random (MNAR). Multiple imputation chained equation (MICE) will be applied if it is deemed appropriate to use the missing at random assumption (MAR).

Data below the limit of quantification (LoQ) for an assay will be substituted with a constant derived by the following equation: LoQ/√2

Ie LOQ divided by the square root of 2.

Loss to follow up and volunteer drop out: the timing and reason of drop out will be recorded and presented in the CONSORT diagram within the appropriate treatment arm.

6.4. ADDITIONAL ANALYSIS

Further exploratory analyses will be conducted if relevant. In the event that non-essential secondary analyses are not available at the time of primary analyses, then analysis of that data will be presented separately.

6.5. HARMS

Adverse events are assessed at regular intervals including at the time of study drug dose titration, and at 3 and 6 months. Volunteers will be asked about gastrointestinal side effects, severe hypoglycaemia, and provided opportunity to disclose other symptoms or hospital presentations. Volunteers are encouraged to contact the study team ad hoc if adverse effects arise outside these scheduled timepoints. The time of ad hoc reviews will be noted in the study file.

Gastrointestinal side effects and severe hypoglycaemia will be coded as binary events (present/ absent since starting study medication).

6.6. STATISTICAL SOFTWARE

IBM SPSS statistical package version 28.0.1.0 will be used. RStudio may be used for select endpoint analysis if SPSS does not offer the required options for generalised linear mixed modelling appropriate to the nature of the data.

7. OTHER

7.1. REFERENCES

1. Gamble C, Krishan A, Stocken D, et al. Guidelines for the Content of Statistical Analysis Plans in Clinical Trials. JAMA 2017;318(23):2337–43.

2. Snaith JR, Samocha-Bonet D, Evans J, et al. Insulin resistance in type 1 diabetes managed with metformin (INTIMET): Study protocol of a double-blind placebo-controlled, randomised trial. Diabetic Medicine. 2021 Sep;38(9):e14564. doi: 10.1111/dme.14564. Epub 2021 Apr 16.

3. ndss-data-snapshot-202103-type1-diabetes.pdf [Internet]. [cited 2021 May 16]; Available from: https://www.ndss.com.au/wp-content/uploads/ndss-data-snapshot-202103-type1-diabetes.pdf

4. Greenfield JR, Samaras K, Chisholm DJ. Insulin Resistance, Intra-Abdominal Fat, Cardiovascular Risk Factors, and Androgens in Healthy Young Women with Type 1 Diabetes Mellitus. J Clin Endocrinol Metab 2002;87(3):1036–40.

5. Schauer IE, Snell-Bergeon JK, Bergman BC, et al. Insulin Resistance, Defective Insulin-Mediated Fatty Acid Suppression, and Coronary Artery Calcification in Subjects With and Without Type 1 Diabetes. Diabetes 2011;60(1):306–14.

6. Wiernsperger NF, Bailey CJ. The antihyperglycaemic effect of metformin: therapeutic and cellular mechanisms. Drugs. 1999;58 Suppl 1:31-9; discussion 75-82. doi: 10.2165/00003495-199958001-00009.

7. Snaith JR, Holmes-Walker DJ, Greenfield JR. Reducing Type 1 Diabetes Mortality: Role for Adjunctive Therapies? Trends in Endocrinology & Metabolism 2020;31(2):150–64.

8. Saghaei M, Saghaei S. Implementation of an open-source customizable minimization program for allocation of patients to parallel groups in clinical trials. Journal of Biomedical Science and Engineering 2011;04(11):734–9.

9. Cree-Green M, Bergman BC, Cengiz E, et al. Metformin Improves Peripheral Insulin Sensitivity in Youth With Type 1 Diabetes. The Journal of Clinical Endocrinology & Metabolism 2019;104(8):3265–78.

10. Wang R, Lagakos SW, Ware JH, Hunter DJ, Drazen JM. Statistics in Medicine — Reporting of Subgroup Analyses in Clinical Trials. New England Journal of Medicine 2007;357(21):2189–94.

11. Twisk J, Bosman L, Hoekstra T, Rijnhard J, Welten M, Heymans M. Different ways to estimate treatment effects in randomised controlled trials. Contemporary Clinical Trials Communications 2018;10:80–5.
