# Supplementary material for: Effect of metformin on insulin resistance in adults with type 1 diabetes: a 26-week randomized double-blind clinical trial
Source: Nat Commun. 2025 Nov 24;16:9884. doi: 10.1038/s41467-025-65951-1 (PMC12644478; doi:10.1038/s41467-025-65951-1)
Supplement: Supplementary file 2 — Reporting Summary [file 41467_2025_65951_MOESM2_ESM.pdf]

nature portfolio

Corresponding author(s): Snaith

Last updated by author(s): Jul 20, 2025

Reporting Summary

Nature Portfolio wishes to improve the reproducibility of the work that we publish. This form provides structure for consistency and transparency in reporting. For further information on Nature Portfolio policies, see our Editorial Policies and the Editorial Policy Checklist.

Statistics

For all statistical analyses, confirm that the following items are present in the figure legend, table legend, main text, or Methods section.

n/a	Confirmed	
☐	☒	The exact sample size (*n*) for each experimental group/condition, given as a discrete number and unit of measurement
☐	☒	A statement on whether measurements were taken from distinct samples or whether the same sample was measured repeatedly
☐	☒	The statistical test(s) used AND whether they are one- or two-sided<br>*Only common tests should be described solely by name; describe more complex techniques in the Methods section.*
☐	☒	A description of all covariates tested
☐	☒	A description of any assumptions or corrections, such as tests of normality and adjustment for multiple comparisons
☐	☒	A full description of the statistical parameters including central tendency (e.g. means) or other basic estimates (e.g. regression coefficient) AND variation (e.g. standard deviation) or associated estimates of uncertainty (e.g. confidence intervals)
☐	☒	For null hypothesis testing, the test statistic (e.g. *F*, *t*, *r*) with confidence intervals, effect sizes, degrees of freedom and *P* value noted<br>*Give P values as exact values whenever suitable.*
☒	☐	For Bayesian analysis, information on the choice of priors and Markov chain Monte Carlo settings
☒	☐	For hierarchical and complex designs, identification of the appropriate level for tests and full reporting of outcomes
☐	☒	Estimates of effect sizes (e.g. Cohen's *d*, Pearson's *r*), indicating how they were calculated

Our web collection on statistics for biologists contains articles on many of the points above.

Software and code

Policy information about availability of computer code

Data collection	Continuous glucose monitoring data were collected using Medtronic iPro ® Enlite system, (Medtronic Northridge, CA) and Dexcom G6 monitor (Dexcom, San Diego, CA) with data downloaded from their commercial systems. No custom code or software was used for other data collection.
Data analysis	Statistical analyses were performed using SPSS version 28.0.1.0 (IBM Corp. Released 2021.). Due to the nature of the data, Rstudio (R Studio 2023.06.1+524 "Mountain Hydrangea"), using the glmmTMB package was used for continuous glucose monitoring data as SPSS did not have the beta-binomial distribution option within its generalized linear mixed modelling framework.

For manuscripts utilizing custom algorithms or software that are central to the research but not yet described in published literature, software must be made available to editors and reviewers. We strongly encourage code deposition in a community repository (e.g. GitHub). See the Nature Portfolio guidelines for submitting code & software for further information.

Data

Policy information about availability of data

All manuscripts must include a data availability statement. This statement should provide the following information, where applicable:

- Accession codes, unique identifiers, or web links for publicly available datasets
- A description of any restrictions on data availability
- For clinical datasets or third party data, please ensure that the statement adheres to our policy

Data from these analyses can my made available upon the condition of compliance with institutional review board restrictions and a data sharing agreement with the project sponsor. The data generated in this study has not been deposited in a public repository due to absence of consent from study participants, and restrictions placed by our study's ethics approval. We encourage researchers or parties interested in collaboration to contact the corresponding author within 5 years of manuscript publication.

Research involving human participants, their data, or biological material

Policy information about studies with human participants or human data. See also policy information about sex, gender (identity/presentation), and sexual orientation and race, ethnicity and racism.

Reporting on sex and gender	The term sex was used in this manuscript. Sixty percent of participants in this study were male, and forty percent were female. For the primary analyses, adjustments for sex were performed. Disaggregated individual level sex data has been collected.
Reporting on race, ethnicity, or other socially relevant groupings	We report ethnicity as % Caucasian, identified by participant self-report.
Population characteristics	In the type 1 diabetes participants, the mean age was 37.4 ± 8.8 years (mean ± SD), with type 1 diabetes duration 22.9 ± 8.9 years, body mass index (BMI) 26.3 ± 3.8 kg/m2, total daily insulin dose 0.6 (0.5, 0.7) units/kg/day (mean [IQR]) and HbA1c 7.5 ± 0.9%. The twenty adults without diabetes were aged 37.0 ± 8.4 years, with BMI 26.2 ± 4.3 kg/m2 and HbA1c 5.1 ± 0.3%. Full demographic details, including self reported ethnicity, family history of type 1 diabetes, prevalence of complications (albuminuria, retinopathy) and usage of multiple daily injections vs insulin pumps are reported in full in table 1 of the manuscript.
Recruitment	Participants were recruited from diabetes clinics at St Vincent's and Westmead Hospitals (academic hospitals in Sydney, Australia), from the private consulting rooms of Endocrinologists, from public advertisements placed on social media, or from word of mouth from other study participants. This strategy was thought to capture a wide demographic of individuals, though would be biased towards metropolitan based people with type 1 diabetes. There was no other anticipated self-selection bias.
Ethics oversight	The study was approved by the St Vincent's Hospital Human Research and Ethics Committee (Sydney)

Note that full information on the approval of the study protocol must also be provided in the manuscript.

Field-specific reporting

Please select the one below that is the best fit for your research. If you are not sure, read the appropriate sections before making your selection.

☒ Life sciences ☐ Behavioural & social sciences ☐ Ecological, evolutionary & environmental sciences

For a reference copy of the document with all sections, see nature.com/documents/nr-reporting-summary-flat.pdf

Life sciences study design

All studies must disclose on these points even when the disclosure is negative.

Sample size	To detect an end-of-treatment difference between groups in EGP of 0.3mg/kg/min with 80% power (presumed 0.3mg/kg/min standard deviation, alpha level 0.05), we determined that we needed 17 participants in each group, increased to 20 to accommodate up to 15% subject drop out.
Data exclusions	A single outlying GDF15 value from the type 1 diabetes group was excluded from the analysis. This datapoint was from an individual who was subsequently diagnosed with a medical condition deemed likely to falsely elevate serum GDF15 levels. Due to medication effects, hormonal contraceptive users were excluded for all analyses involving sex-hormone binding globulin. This is is reported in the manuscript.
Replication	The findings were not replicated, as this was a randomized controlled trial and replication would require conduct of a second trial.
Randomization	Randomization was performed by a designated independent clinician who was uninvolved with the study, then provided the allocation to the designated unblinded study pharmacist. Randomization was 1:1 for metformin to placebo using a computerized minimization procedure to stratify for BMI, HbA1c, gender and age (40%, 20%, 20% and 20% weighting respectively)

Blinding | The study was double-blinded. Participants, outcome assessors and data analysts were blinded to treatment allocation. Metformin and placebo capsules and their packaging were identical in appearance.

Reporting for specific materials, systems and methods

We require information from authors about some types of materials, experimental systems and methods used in many studies. Here, indicate whether each material, system or method listed is relevant to your study. If you are not sure if a list item applies to your research, read the appropriate section before selecting a response.

Materials & experimental systems

n/a	Involved in the study
☒	☐ Antibodies
☒	☐ Eukaryotic cell lines
☒	☐ Palaeontology and archaeology
☒	☐ Animals and other organisms
☐	☒ Clinical data
☒	☐ Dual use research of concern
☒	☐ Plants

Methods

n/a	Involved in the study
☒	☐ ChIP-seq
☒	☐ Flow cytometry
☒	☐ MRI-based neuroimaging

Clinical data

Policy information about clinical studies

All manuscripts should comply with the ICMJE guidelines for publication of clinical research and a completed CONSORT checklist must be included with all submissions.

Clinical trial registration | The trial was registered with the Australian New Zealand Clinical Trials Registry (ANZCTR). Registration number: ACTRN12619001440112.

Study protocol | The trial protocol was published, and available from DOI: 10.1111/dme.14564.

Data collection | All data was collected at the Garvan Institute of Medical Research, between November 2019 and December 2021.

Outcomes | The primary endpoint was change in liver insulin sensitivity after 26 weeks, defined by endogenous glucose production during the low-dose insulin phase of the hyperinsulinemic-euglycemic clamp. Pre-specified secondary endpoints included the change in muscle insulin sensitivity (defined by glucose infusion rate during the high-dose insulin phase of the hyperinsulinemic euglycemic clamp), change in adipose insulin sensitivity (defined by the non-esterified fatty acid (NEFA) levels during the low-dose hyperinsulinemic-euglycemic clamp), total daily insulin dose (assessed by dosing diary or insulin pump download), continuous glucose monitoring parameters, serum or plasma measures (Hba1c, GDF15, lipids, uric acid, sICAM-1, IL-6, sE-selectin, IGF-1, adiponectin, augmentation index (assessed by radial artery application tonometry), body composition (fat mass, fat free mass, visceral adipose tissue, by DXA scan, anterior thigh muscle fat infiltration, abdominal subcutaneous adipose tissue volume by MRI, controlled attenuation parameter by transient elastography, anthropometrics (body weight by scale, blood pressure by sphygmanometry, waist circumference by tape measure).

Glucagon and dietary intake analyses were exploratory, and other secondary endpoints including microbiome analyses are not reported in this article.

Plants

Seed stocks | NA

Novel plant genotypes | NA

Authentication | NA
